# The Role of Various Types of Diets in the Treatments of Depressive Disorders

**DOI:** 10.3390/medicina61101737

**Published:** 2025-09-24

**Authors:** Anna Lis, Patrycja Maj, Agata Świętek, Ewa Romuk

**Affiliations:** 1 Students Research Team at the Department of Biochemistry, Faculty of Medical Sciences in Zabrze, Medical University of Silesia, 19, Jordan St., 41-808 Zabrze, Poland; s88013@365.sum.edu.pl (A.L.); s88241@365.sum.edu.pl (P.M.); 2 Department of Medical and Molecular Biology, Faculty of Medical Sciences in Zabrze, Medical University of Silesia in Katowice, 19, Jordan St., 41-808 Zabrze, Poland; agata.swietek@sum.edu.pl; 3 Silesia LabMed Research and Implementation Center, Medical University of Silesia in Katowice, 19, Jordan St., 41-808 Zabrze, Poland; 4 Department of Biochemistry, Faculty of Medical Sciences in Zabrze, Medical University of Silesia, 19, Jordan St., 41-808 Zabrze, Poland

**Keywords:** depression theories, gut microbiome, Mediterranean diet, anti-inflammatory diet, ketogenic diet, Western diet

## Abstract

Depression is a prevalent and disabling psychiatric disorder, characterized by persistent disturbances in mood, cognition, and physiological processes, which collectively lead to substantial impairments in daily functioning and quality of life. This review provides a comprehensive overview of the biological mechanisms implicated in the pathophysiology of depression, including neurotransmitter dysregulation, oxidative stress, inflammatory processes, hypothalamic-pituitary-adrenal (HPA) axis dysfunction, mitochondrial impairment, and alterations in the gut-brain axis. Furthermore, it explores the role of diet in both the prevention and management of depression, with particular emphasis on Mediterranean, anti-inflammatory, and ketogenic dietary patterns, while contrasting these with the detrimental impact of a Western dietary pattern. Specific nutrients-such as n-3 polyunsaturated fatty acids (PUFAs), B-complex vitamins, vitamins D and E, zinc, selenium, and polyphenols-are highlighted for their potential roles in modulating neurotransmission, attenuating inflammation, and supporting gut microbiota homeostasis. Despite growing scientific interest in nutrition-based interventions, current evidence on the comparative efficacy of different dietary approaches remains limited. Future research is warranted to elucidate the therapeutic potential of dietary strategies as adjuncts to conventional treatments for depression and to facilitate the development of evidence-based nutritional recommendations for clinical practice.

## 1. Introduction

Depressive disorders represent one of the most prevalent psychiatric conditions worldwide. According to the International Classification of Diseases, 11th Revision (ICD-11), they are defined by the presence of depressed mood or loss of pleasure, accompanied by a constellation of cognitive, emotional, and somatic symptoms that result in clinically significant distress or impairment across personal, social, educational, occupational, and other domains of functioning. The World Health Organization estimates that approximately 280 million individuals are currently affected by depression, with the disorder contributing to nearly 700,000 deaths annually and ranking as the fourth leading cause of mortality among individuals aged 15–29 years [1]. In Poland, data from the National Health Fund indicate that nearly 1.2 million individuals are living with depression [2].

Depression profoundly impacts thoughts, emotions, behavior, and overall well-being [3]. Clinically, it is characterized by a persistently depressed or deteriorating mood, anhedonia, reduced concentration, fatigue, diminished psychomotor activity, sleep disturbances, and frequently low self-esteem [4,5]. According to the Diagnostic and Statistical Manual of Mental Disorders, Fifth Edition (DSM-5), a major depressive episode is diagnosed when an individual experiences a depressed mood or loss of interest/pleasure for at least two weeks, in combination with additional psychological or somatic symptoms, resulting in a marked decline from previous functioning [6].

The etiology of depressive disorders is increasingly recognized as multifactorial and complex. While neurotransmitter dysregulation plays a central role, other mechanisms include disturbances of the gut-brain axis mediated by intestinal dysbiosis, HPA axis dysfunction, chronic oxidative stress, increased inflammatory activity, and mitochondrial impairment [7,8,9,10,11,12]. Risk factors encompass both genetic predispositions and environmental influences. Social determinants, such as isolation, lack of supportive relationships, and persistent interpersonal stress, are strongly associated with elevated risk and greater severity of depression [13]. Emotional regulation difficulties, including maladaptive rumination and emotional suppression, may exacerbate symptomatology and impede recovery [14]. Moreover, behavioral factors such as reduced engagement in rewarding activities, sedentary lifestyle, and disrupted sleep patterns further contribute to the onset and persistence of depressive episodes. Psychosocial interventions targeting these domains may significantly enhance treatment outcomes [15].

Management of depression requires an integrative approach that combines pharmacotherapy, psychotherapy, and lifestyle modifications, including structured physical activity and dietary interventions. Pharmacological treatments, most commonly based on antidepressants such as selective serotonin reuptake inhibitors (SSRIs) and monoamine oxidase inhibitors (MAOIs), act by modulating neurotransmitter availability [16]. Psychotherapeutic approaches-particularly cognitive behavioral therapy (CBT) and interpersonal therapy (IPT)-help patients restructure maladaptive cognitions and improve social functioning [17].

Growing attention is directed toward the role of diet in the prevention and management of depression. Epidemiological and clinical studies suggest that dietary patterns can influence both the risk of developing depression and the course of the disorder in affected individuals [18]. Nutrients such as n-3 PUFAs, B vitamins, magnesium, zinc, and antioxidants are of particular relevance due to their capacity to modulate neurotransmission and attenuate systemic inflammation. Diets characterized by high intakes of plant-based foods (vegetables, fruits, legumes) and fish appear to exert protective effects, whereas dietary patterns rich in highly processed foods are associated with elevated risk [19].

**Figure 1:**
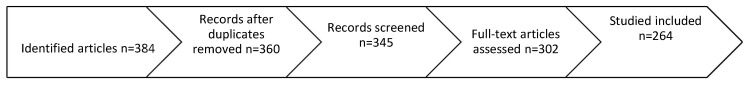
PRISMA-style flow diagram.

Although prior research has predominantly focused on individual nutrients or single dietary patterns, few studies have directly compared multiple dietary approaches in the context of depression. This review seeks to address this gap by systematically analyzing and contrasting different dietary patterns, with the aim of identifying those that may provide the most effective preventive and therapeutic benefits. By synthesizing current evidence, the review not only summarizes existing findings but also evaluates their relative efficacy, thereby offering a hypothesis-driven perspective that extends beyond prior general overviews. For greater clarity, we have included a PRISMA-style flow diagram (Figure 1), table summarizing the inclusion and exclusion criteria (Table 1), tables of randomized controlled trials (RCTs) (Table 2) and summary table illustrating the impact of different type of diets on depressive disorders (Table 3).

Explicit search strategy:

The databases PubMed, Embase, Web of Science, Scopus, MEDLINE, and Cochrane CENTRAL were searched up to August 2025. The search was limited to publications in Polish and English. Keywords were used including: ‘depression’ OR ‘depressive disorder’ OR ‘mood’ OR ‘mental health’ AND ‘oxidative’ OR ‘inflammatory’ OR ‘vitamin B’ OR ‘vitamin D’ or ‘vitamin E’ OR ‘mitochondria’ AND ‘mediterranean’ OR ‘ketogenic’ OR ‘vegetarian’ OR ‘western’ AND ‘gut microbiome’ AND ‘monoamine’ AND ‘HPA axis’ AND ‘randomized controled trial’ AND.

**Table 2:** Randomized Controlled Trials table (RCT).

Intervention	Studies	Population	Duration (Range)	Main Findings
Dietary improvement (SMILES trial)	[19]	Adults with major depression	12 weeks	Improved depressive symptoms vs. control
Zinc + imipramine	[20]	Patients with depression	6 weeks	Zinc enhanced antidepressant response
Selenium	[21]	Healthy adults	6 weeks	Mood improvement
Coenzyme Q10	[22]	Patients with bipolar depression	8 weeks	Improvement in depressive symptoms
Folate	[23]	Individuals with depression	Protocol only	RCT planned; no results reported
n-3 PUFAs	[24]	Patients with depression + CVD	12 weeks	Improvement in depressive symptoms in omega-3 group
Anti-inflammatory dietary education	[25]	Depressed breast cancer patients on chemotherapy	12 weeks	Reduction in depressive symptoms
Vitamin D	[26]	Patients with depression	8 weeks	Improvement in depressive symptoms and neurotransmitter levels
Resveratrol (±piperine/+equol)	[27,28,29]	Healthy adults; menopausal and postmenopausal women	28 days–14 weeks	Improved cognition, cerebral blood flow, quality of life, and mood
Curcumin (incl. add-on therapy)	[30,31]	Patients with major depression	6–8 weeks	Significant or additional improvement in depressive symptoms

**Table 3:** Role of various type of diets in depressive disorders.

Aspect	Mediterranean	Anti-Inflammatory	Ketogenic	Western	Vegetarian
Influenced mechanism	Anti-inflammatory, supports neurotransmission, gut microbiota	Lowers pro-inflammatory cytokines, antioxidant effects	Ketone bodies, GABA modulation, mitochondrial support	Promotes inflammation, oxidative stress, HPA dysregulation	High antioxidants, risk of B12, iron, n-3 deficiencies
Products	Olive oil, vegetables, fish, nuts, whole grains	Fatty fish, nuts, fruits, vegetables, tea, turmeric	High-fat (avocado, nuts, oils), moderate protein, low-carb vegetables	Fried/processed foods, sugary drinks, sweets	Legumes, vegetables, fruits, nuts, whole grains; dairy/eggs (lacto-ovo), fish (pesco)
Biological effects	Reduces inflammation, improves neurotransmitter synthesis	Reduces oxidative stress, improves mood	Enhances mood stability, reduces anxiety/depression-like behaviors	Increases depression risk, reduces hippocampal plasticity	May reduce inflammation; deficiencies can increase risk
Clinical evidence	Cohort and RCTs show lower depression risk and symptoms	Small trials show symptom improvement	Animal and early human studies show benefit	Consistently linked to higher risk of depression	Mixed results: some cohorts show lower risk, others higher
Future research	Longitudinal and supplementation trials (vitamin D, B12, omega-3)	Larger RCTs on polyphenols, vitamins D/E; combination strategies	Long-term safety, efficacy, mechanistic studies in humans	Human intervention studies to clarify causality	Clarify impact of nutrient deficiencies; long-term trials

## 2. Monoamine Theory of Depression

The monoamine theory remains one of the principal hypotheses explaining the pathophysiology of depression. It postulates that deficits in monoamine neurotransmitters, such as serotonin (5-HT), dopamine (DA), and norepinephrine (NE), underlie depressive symptomatology. However, this model does not fully account for the neurobiological and molecular complexity of depression nor does it adequately explain the mechanisms of action of psychotropic drugs [32].

5-HT is synthesized from dietary tryptophan via the intermediate metabolite 5-hydroxytryptophan in the dorsal raphe nucleus. This process is highly dependent on plasma tryptophan availability, which is influenced by nutritional intake [33]. In individuals with an increased risk of developing depression, tryptophan restriction or deficiency can exacerbate or trigger symptoms [34,35]. The 5-HT hypothesis emphasizes reduced serotonergic transmission, accompanied by receptor dysregulation-upregulation of 5-HT2 receptors and downregulation of 5-HT1A receptor expression and sensitivity-resulting in impaired postsynaptic signaling [36]. Depressed individuals frequently exhibit decreased serum 5-HT and plasma tryptophan levels, along with altered concentrations of 5-HT metabolites (e.g., 5-hydroxyindoleacetic acid (5-HIAA)) in cerebrospinal fluid [37]. While serotonergic antidepressants such as tricyclic antidepressants (TCAs), SSRIs, and serotonin-norepinephrine reuptake inhibitors (SNRIs) aim to enhance synaptic 5-HT availability, their efficacy may be influenced by nutritional status, particularly levels of vitamin B6, folate, and iron, which act as essential cofactors in 5-HT biosynthesis [38].

NE synthesis begins with dietary phenylalanine, which is converted to tyrosine, then to DA, and subsequently to NE via dopamine β-hydroxylase. Low activity of this enzyme, often associated with stress, shifts catecholamine synthesis toward DA rather than NE [39]. Noradrenergic dysfunction in depression includes reduced norepinephrine transporter (NET) density and increased α2-adrenergic receptor expression in the locus coeruleus [40,41]. Adequate biosynthesis of NE requires not only precursor amino acids but also vitamin C and copper as enzymatic cofactors, and deficiencies may contribute to impaired neurotransmission and diminished response to noradrenergic antidepressants [39].

DA plays a pivotal role in attention, motivation, reward, motor function, and emotional regulation [42]. The e hypothesis arose from observations linking psychomotor retardation in depression to reduced dopaminergic activity [43]. Chronic stress disrupts reward-related circuits, contributing to fatigue and anhedonia [44]. Lower levels of DA metabolites in cerebrospinal fluid have also been reported in depressed patients [45]. As with other monoamines, DA synthesis depends on adequate dietary tyrosine and enzymatic cofactors, including vitamin B6 and iron; deficiencies in these nutrients may perpetuate dopaminergic dysregulation.

Other neurotransmitters, including γ-aminobutyric acid (GABA), glutamate, and histamine, are increasingly recognized in depression pathophysiology [46,47]. Histamine, synthesized in tuberomammillary neurons, influences arousal, cognition, and stress regulation. Through interactions with H3 and H4 receptors, histamine modulates serotonergic, dopaminergic, and noradrenergic signaling. Preclinical studies suggest that stress and inflammation alter histaminergic tone, and H3 receptor antagonists may exert antidepressant effects by restoring neurogenesis. SSRIs may also modulate histamine dynamics, underscoring possible interplay between antidepressant mechanisms and histaminergic signaling [48].

Despite its historical prominence, the monoamine theory has faced increasing criticism. Antidepressants developed under this model are limited by delayed onset of therapeutic effects, treatment resistance, and their inability to explain the primary causes of neurotransmitter imbalance. For example, Moncrieff et al. (2023) reviewed 5-HT-related research and reported insufficient evidence linking depression to reduced 5-HT levels or activity, thereby questioning the rationale for widespread antidepressant use. However, these conclusions have been contested due to methodological concerns and misinterpretations of pharmacological and neuroimaging data [49].

Nevertheless, monoamines are implicated in broader neurobiological processes beyond neurotransmission. They regulate microglial activation, cytokine release [50], synaptic plasticity, brain-derived neurotrophic factor (BDNF) signaling, and HPA axis activity through glucocorticoid receptor modulation [51]. Chronic monoamine deficiency may impair neuroprotection, reduce hippocampal neurogenesis [52], and attenuate BDNF signaling [53]. In addition, it may destabilize HPA axis feedback, exacerbate cortisol-induced neurotoxicity, and promote neuronal injury, all of which contribute to the development and persistence of depression [54].

## 3. The Oxidative Theory of Depression

The oxidative stress hypothesis of depression proposes that a chronic imbalance between the excessive generation of reactive oxygen species (ROS) and the efficiency of antioxidant defense mechanisms contributes to neuronal damage and depressive symptomatology. Oxidative stress disrupts the integrity of lipids, proteins, and DNA within the central nervous system, thereby impairing synaptic transmission, weakening neuroplasticity, and disturbing neurotransmitter signaling pathways [55,56,57]. Oxidative stress is defined as an imbalance between ROS production and the organism’s antioxidant capacity. Two principal defense systems are responsible for ROS elimination: non-enzymatic antioxidants, including vitamins C and E, coenzyme Q10, carotenoids, glutathione, and trace elements; and enzymatic antioxidants such as superoxide dismutase (SOD), catalase (CAT), glutathione peroxidase (GPx), glutathione reductase (GR), and glutathione S-transferase (GST) [58,59,60,61]. ROS exert their detrimental effects through mechanisms such as lipid peroxidation, protein oxidation, and mitochondrial dysfunction. For instance, lipid peroxidation, particularly of polyunsaturated fatty acids, has been extensively documented [62]; protein oxidation disrupts enzymatic homeostasis [63]; and oxidative modifications of mitochondrial proteins impair energy metabolism [64]. The brain is particularly vulnerable to oxidative injury due to its high oxygen demand, abundance of polyunsaturated fatty acids, presence of redox-active transition metals, and relatively limited antioxidant defenses [65]. Clinical studies support this hypothesis: patients with depression demonstrate elevated levels of malondialdehyde (MDA), a biomarker of lipid peroxidation, with higher concentrations observed in recurrent versus first-episode depression [10,66]. Similarly, 8-iso-prostaglandin F2α, a marker of arachidonic acid peroxidation, and 8-oxoguanine, indicative of oxidative DNA damage, have been reported at increased levels in depressed individuals [67,68]. Deficits in antioxidant defenses further support this theory. Patients with depression exhibit decreased concentrations of zinc, glutathione, coenzyme Q10, and vitamins A, C, and E, alongside increased activity of pro-oxidant enzymes such as xanthine oxidase [69,70,71]. Zinc, an essential cofactor for antioxidant enzymes such as SOD, has been consistently implicated; a meta-analysis revealed that peripheral zinc levels are, on average, 1.850 µmol/L lower in depressed patients compared with controls [72,73]. Reduced zinc levels correlate with symptom severity and impaired glutamatergic regulation in the hippocampus and cortex, thereby compromising neuroplasticity and cognitive function [20,74,75,76,77,78,79,80]. Selenium is another critical component of antioxidant defense, primarily through its role in GPx activity and the regeneration of vitamins C and E [81]. Clinical trials have demonstrated that selenium supplementation improves mood and reduces anxiety, whereas selenium deficiency is associated with increased depressive symptoms [21,82,83,84,85]. However, both insufficient and excessive selenium levels may disrupt oxidative-inflammatory balance, with low selenium correlating with elevated pro-inflammatory cytokines such as IL-6 and CRP [86,87,88,89]. Moreover, selenium modulates dopaminergic, serotonergic, and noradrenergic systems, highlighting its multifaceted role in depression [90,91]. Emerging interest has focused on antioxidant-based interventions. Ebselen, an organoselenium compound with GPx-mimetic activity, demonstrates antioxidant and neuroprotective properties by inhibiting inositol monophosphatase (IMPase) and modulating glutamatergic signaling [92]. However, in clinical studies of treatment-resistant depression, ebselen did not produce significant improvements in depressive symptoms [93]. In summary, oxidative stress appears to play a central role in the pathophysiology of depression. While evidence supports the association between oxidative damage, reduced antioxidant defenses, and depressive symptoms, the therapeutic efficacy of antioxidant-based strategies remains inconclusive. Future long-term, well-controlled trials are necessary to clarify the clinical relevance of targeting oxidative stress in depression.

## 4. The Inflammatory Theory of Depression

The inflammatory hypothesis of depression proposes that activation of the immune system contributes to the onset and progression of depressive disorders. Patients with depression frequently exhibit increased numbers of circulating neutrophils, macrophages, and T lymphocytes, along with elevated plasma levels of prostaglandin E2 and pro-inflammatory cytokines, including interleukin IL-1β, IL-6, interferon-γ (IFN-γ), and tumor necrosis factor-α (TNF-α) [94,95]. The link between immune dysregulation and depression was first highlighted by Maes in 1999, who demonstrated associations between depressive symptomatology and immune activation [96]. Subsequent studies have confirmed a higher prevalence of depression in conditions characterized by acute or chronic inflammation, such as autoimmune and infectious diseases [11,97]. Pro-inflammatory cytokines exert multiple effects relevant to depression pathophysiology. Elevated cortisol levels, secondary to cytokine activity, can impair hippocampal function and disrupt neuroendocrine balance [98]. Cytokines also influence neurotransmitter metabolism by enhancing indoleamine 2,3-dioxygenase (IDO) activity, which diverts tryptophan metabolism from 5-HT synthesis toward the kynurenine pathway. This process increases the production of neurotoxic metabolites such as quinolinic acid, thereby reducing serotonergic signaling and exacerbating depressive symptoms [99,100]. Inflammatory activity additionally contributes to cognitive dysfunction in depression. Elevated cytokine levels have been associated with deficits in attention, memory, and executive functioning, likely mediated through impaired neuroplasticity, increased oxidative stress, and subsequent neuronal injury [99]. Moreover, chronic inflammation decreases the expression of BDNF, further weakening synaptic plasticity and reducing the adaptive capacity of neural circuits involved in mood regulation [101].

## 5. The Role of Mitochondria in Maintaining Oxidative-Inflammatory Balance in the Body

Mitochondria are the primary energy-generating organelles in eukaryotic cells, producing adenosine triphosphate (ATP) via oxidative phosphorylation. This function is particularly critical in the brain, which exhibits high energy demand and contains large numbers of mitochondria localized within dendrites and synapses. A single cortical neuron at rest is estimated to consume approximately 4.7 billion ATP molecules per second [102,103,104]. Beyond energy metabolism, mitochondria regulate apoptosis and act as major sources of ROS, thereby playing a central role in cellular redox balance. Growing evidence suggests that mitochondrial dysfunction contributes to the pathogenesis of depression [12]. Individuals with depression frequently display impaired bioenergetics, reflected in reduced ATP synthesis, which may underlie symptoms such as fatigue and psychomotor retardation [105]. Elevated oxidative stress further compromises mitochondrial integrity, while alterations in mitochondrial DNA (mtDNA) copy number and increased mtDNA damage disrupt protein synthesis and impair organelle function [106,107]. Chronic inflammation, commonly observed in depression, may also impair mitochondrial activity. Pro-inflammatory cytokines, including IL-1β, IL-6, and TNF-α, can suppress ATP production and enhance ROS generation, amplifying oxidative–inflammatory imbalance [106]. Additionally, chronic stress, mediated by glucocorticoid excess, adversely affects mitochondrial function and promotes neuronal vulnerability [105]. Endogenous antioxidants such as glutathione (GSH), coenzyme Q10 (CoQ10), and alpha-lipoic acid (ALA) are critical for counteracting mitochondrial dysfunction. GSH serves as the principal intracellular antioxidant, neutralizing ROS generated in mitochondria. Reduced GSH levels, reported in the cerebral cortex and occipital regions of patients with depression, contribute to lipid, protein, and DNA damage, ultimately impairing neuronal efficiency [108,109,110]. Similarly, CoQ10, an essential component of the electron transport chain, prevents electron leakage and limits ROS formation. Depressed individuals often exhibit reduced plasma CoQ10 concentrations, which correlate with symptom severity and treatment resistance. Supplementation with CoQ10 has shown therapeutic benefits in depression and related disorders, including bipolar disorder and chronic fatigue syndrome [22,111,112]. ALA, functioning both as an antioxidant and as a mitochondrial enzyme cofactor, regenerates other antioxidants (e.g., GSH, vitamins C and E) and enhances neuronal redox stability. Clinical studies indicate that ALA supplementation reduces depressive symptoms, lowers inflammatory markers such as C-reactive protein (CRP), and decreases oxidative damage, suggesting neuroprotective potential [113,114].

## 6. The Role of the Gut-Brain Axis in Depression

The gut-brain axis is a bidirectional communication system linking the gut microbiota with the central nervous system (CNS) [115,116]. This complex network involves the enteric and autonomic nervous systems, neuroendocrine signaling, and immune mechanisms [117]. Direct communication occurs via the vagus nerve, whereas indirect signaling is mediated through endocrine and immune pathways. The gut microbiota can influence brain function by modulating vagal activity, producing neuroactive compounds, fermenting dietary substrates, and regulating hormone secretion [118]. The gut microbial community is predominantly composed of bacteria, with smaller contributions from fungi, viruses, archaea, and protozoa. Its composition is shaped early in life by perinatal factors and subsequently modulated by environmental exposures, diet, physical activity, and overall health status. Among these factors, diet represents the most modifiable determinant, with specific dietary patterns capable of steering the microbiota toward either pro-inflammatory or neuroprotective configurations [119,120]. The gut microbiota contributes to intestinal homeostasis by regulating digestion, luminal pH, and nutrient absorption [115]. In addition, microorganisms synthesize vitamins (e.g., vitamin K and B-group vitamins) and produce short-chain fatty acids (SCFAs) through fiber fermentation, which exert anti-inflammatory and neuroprotective effects [115,121,122]. The microbiota also strengthens the intestinal barrier by maintaining epithelial integrity and preventing pathogen translocation [123]. Immune regulation is another key function, with modulation of cytokine levels occurring via interactions with gut-associated lymphoid tissue [115]. Furthermore, microbial metabolites influence the secretion of hormones such as peptide YY (PYY), glucagon-like peptide-1 (GLP-1), and cholecystokinin (CCK), thereby affecting appetite regulation and glucose homeostasis [124]. Gut microbiota also modulate CNS activity by regulating serotonergic, noradrenergic, dopaminergic, glutamatergic, and GABAergic neurotransmission through their effects on neurotransmitter synthesis and metabolism. In addition, they participate in tryptophan metabolism, directing it toward 5-HT, kynurenine, or indole derivatives-all of which are implicated in mood regulation and cognitive processes [117]. Gut dysbiosis, defined as an imbalance in microbial composition, has been consistently associated with psychiatric conditions including schizophrenia and depression [125,126]. Stress exposure significantly alters microbiota composition and abundance. In humans, stress reduces the levels of *Lactobacillus* spp. and *Bifidobacterium* spp., whereas in rodents stress decreases *Lactobacillus* abundance and increases *Clostridium* spp. Catecholamine release further promotes the growth of *Escherichia coli*, both pathogenic and non-pathogenic. These findings support the strong link between stress, gut microbiota alterations, and the onset of mood disorders [115]. A key mechanism involves increased intestinal permeability (“leaky gut syndrome”), which facilitates the translocation of Gram-negative bacteria and lipopolysaccharides (LPS) into the circulation, thereby triggering systemic inflammation. Patients with depression exhibit elevated IgM and IgA antibodies against LPS from gut-resident Gram-negative bacteria, consistent with enhanced gut permeability and immune activation [127]. Microbiome analyses have revealed reduced microbial diversity and richness in individuals with depression, characterized by increased abundance of potentially pro-inflammatory genera (*Blautia*, *Klebsiella*, *Clostridium*) and decreased abundance of beneficial taxa such as *Faecalibacterium*, *Bifidobacterium*, and *Ruminococcus* [124]. Evidence from germ-free (GF) animal models further supports a causal role of the microbiota in emotional regulation and neurodevelopment. GF mice display alterations in neurogenesis, myelination, stress responsivity, and neurotransmitter levels, including 5-HT and BDNF expression in the hippocampus and prefrontal cortex [128].

## 7. HPA Axis Dysregulation

A well-established relationship exists between stress and depression, with stress serving as a potent precipitating factor for depressive episodes, particularly in stress-vulnerable individuals [129]. Childhood adversity, including physical or emotional abuse and neglect, further increases the risk of depression in adulthood [130]. In animal models, chronic mild stress induces behavioral changes resembling depressive symptoms, including reduced sucrose preference, diminished sexual activity, decreased motivation, neglect of grooming, and sleep disturbances [131].

The HPA axis, a central component of the neuroendocrine stress response system, is consistently implicated in the pathophysiology of depression [132]. Upon stress exposure, the paraventricular nucleus (PVN) of the hypothalamus releases corticotropin-releasing hormone (CRH), which stimulates the anterior pituitary to secrete adrenocorticotropic hormone (ACTH). ACTH then induces glucocorticoid (GC) release, primarily cortisol, from the adrenal cortex [133,134]. Under physiological conditions, a negative feedback loop involving glucocorticoid receptors (GRs) in the hypothalamus, hippocampus, and prefrontal cortex inhibits CRH release, thereby preventing excessive cortisol accumulation [135].

Chronic or severe stress may disrupt this feedback regulation, leading to sustained glucocorticoid hypersecretion and maladaptive neurobiological changes [136]. Structural brain alterations, including reduced hippocampal and prefrontal cortex volumes, have been documented in depression and are associated with dendritic retraction and synaptic dysfunction [137]. Dysfunction of the nucleus accumbens, a key component of the brain’s reward system, may contribute to anhedonia frequently observed in depressed patients [138]. Excess glucocorticoids promote excitotoxicity, impair neurogenesis in the dentate gyrus of the hippocampus, and enhance neuronal vulnerability to additional insults such as hypoxia, hypoglycemia, and seizures [139,140].

Two major receptor types mediate cortisol activity in the CNS: mineralocorticoid receptors (MRs), which regulate basal HPA activity, and GRs, which mediate feedback inhibition during stress. Abnormal expression or reduced density of GRs in the hippocampus and prefrontal cortex has been observed in depression, contributing to impaired HPA axis feedback and chronic hypercortisolemia [136]. Preclinical studies demonstrated that chronic stress reduces GR density in the hippocampus, whereas long-term antidepressant treatment increases GR expression. These findings support the hypothesis that GR dysfunction represents a key mechanism underlying HPA axis dysregulation in depression [141].

## 8. The Mediterranean Diet in the Treatment of Depression

The Mediterranean diet (MedDiet), first described by Ancel Keys in 1960 [142], is one of the most extensively studied and widely recognized dietary patterns globally. Traditionally, it emphasizes high consumption of plant-based foods, including fruits, vegetables, minimally processed cereals, potatoes, legumes, nuts, and seeds. Olive oil serves as the primary source of fat, while dairy products (mainly cheese and yogurt) and fish or poultry are consumed in moderation. Red meat is limited, and sweets containing sugar or honey are consumed sparingly. Moderate wine intake with meals is also characteristic of this dietary pattern [143]. The MedDiet has been associated with beneficial effects in depression, with specific components contributing to these outcomes [144]. It supports gut microbial diversity and stability, increasing the abundance of *Faecalibacterium prausnitzii*, *Bifidobacterium*, and *Lactobacillus*, while reducing pro-inflammatory taxa such as *Proteobacteria*. These alterations are linked to enhanced production of SCFAs, reduced intestinal permeability, and lower systemic inflammation, all of which may contribute to its antidepressant effects [145]. The MedDiet ensures adequate intake of B vitamins, whose deficiency can lead to homocysteine accumulation and impaired synthesis of key neurotransmitters involved in mood regulation [146,147]. Olive oil provides eicosapentaenoic acid (EPA), which possesses anti-inflammatory and neuroprotective properties [148]. Polyphenols present in the diet modulate gut microbiota and exert anti-inflammatory effects [149]. High fiber intake promotes microbial diversity and is associated with a lower risk of depression [150]. Vegetables supply α-tocopherol, B vitamins, carotenoids, vitamin C, and magnesium, while legumes provide tryptophan, a precursor of 5-HT, as well as zinc, which functions as an NMDA receptor antagonist, modulating glutamatergic signaling [151]. These plants also contain zinc, which acts as an NMDA receptor antagonist, inhibiting glutamate binding. The MedDiet is believed to be beneficial in the treatment of depression, with specific components contributing to its positive effects [144]. In a study by Masana et al. [152], strict adherence to the Mediterranean diet was associated with a 35% reduction in the risk of developing depressive symptoms. A meta-analysis by Psaltopoulou et al. [153] confirmed that high compliance with this dietary pattern reduces the risk of depression across different age groups. The diet also provides folic acid and vitamin B12, both critical for neurotransmitter synthesis and antidepressant response. Reduced erythrocyte concentrations of these vitamins have been observed in depressed patients [23,154]. Folic acid deficiency is associated with impaired synthesis of DA, NE, and 5-HT and may decrease pharmacotherapy efficacy [23,155]. Adequate vitamin B12 intake during treatment may enhance antidepressant responsiveness, although comprehensive studies assessing its precise impact remain limited [156]. However, there is still a lack of comprehensive studies regarding vitamin B12 supplementation in depression and its precise impact on the disorder. Hibbeln et al. published findings showing that increased fish consumption was correlated with a reduced annual incidence of depression globally [156]. Numerous studies have demonstrated that a MedDiet diet rich in polyphenols alleviates and helps prevent depressive symptoms [24,157]. Individuals following mediterranean diet often engage more in communal meals and family-oriented eating habits, which promote social bonding and a sense of belonging, both of which both of which serve as protective factors for mental health, including depression [158]. Despite being one of the most well-researched dietary patterns, there is still a lack of comprehensive studies regarding vitamin B12 supplementation in depression and its precise impact on the disorder. Further studies are needed to evaluate the efficacy of the MedDiet as a therapeutic strategy for depression. Future research should include large randomized controlled trials to assess both overall adherence and the contribution of specific components such as olive oil, polyphenols, n-3 PUFAs, dietary fiber, and B vitamins. Long-term effects, population differences, and potential interactions with pharmacological or psychotherapeutic treatments also warrant further investigation.

## 9. Anti-Inflammatory Diet in the Treatment of Depression

Dietary habits play a critical role in regulating systemic inflammation, which is increasingly recognized as a factor in the pathophysiology of depression. Pro-inflammatory diets contribute to elevated inflammatory responses, whereas anti-inflammatory dietary patterns may mitigate these processes [159,160]. To quantify the inflammatory potential of diet, the Dietary Inflammatory Index (DII) has been developed [161].

Anti-inflammatory diets are typically rich in nutrients with anti-inflammatory properties, including n-3 PUFAs [162], zinc [163], polyphenols [164], and vitamins, particularly D and E [165,166]. EPA, a key n-3 PUFA, serves as a precursor for bioactive compounds such as prostaglandins and leukotrienes that are essential for optimal brain function [167]. Clinical studies suggest that EPA supplementation may improve depressive symptoms more effectively than docosahexaenoic acid (DHA) or combined n-3 PUFA supplementation [168]. The neuroprotective effects of n-3 PUFAs are attributed to their structural role in neuronal myelin sheaths and their regulation of neurotransmitter flow and synaptic signaling [169]. Reduced intake of n-3 PUFAs has been associated with increased prevalence of depression [155,167], and supplementation may benefit individuals with elevated oxidative stress [170]. Findings by H. Bakhtiari-Dovvombaygi et al. suggest that vitamin D is a nutrient that significantly reduces anxiety and depressive symptoms by inhibiting neuroinflammation and reducing oxidative stress [171]. Research by A. Koshkina et al. demonstrated that high doses of vitamin D3 exhibit antidepressant effects and counteract anhedonia. These outcomes may be linked to the vitamin’s role in regulating the expression of neurotrophic factors such as nerve growth factor (NGF), glial cell line-derived neurotrophic factor (GDNF), and neurotrophin-3 (NT-3). The authors also propose that vitamin D3 modulates cAMP response element-binding protein (BDNF-CREB) signaling and helps restore balance in the HPA axis, which is often dysregulated in stress-induced depressive states [172]. However, further research is necessary to fully elucidate how vitamin D3 influences BDNF expression in the hippocampus. Additionally, vitamin D appears to have a protective effect on the dopaminergic system [173] and plays a neuroprotective role in neuronal proliferation, differentiation, survival, and growth [174,175]. M. Kaviani et al., studying adults with mild to moderate depression, demonstrated reduced severity of depressive symptoms following vitamin D3 supplementation [26]. Vitamin E comprises a group of fat-soluble organic compounds, including tocopherols (T) and tocotrienols (T3). It functions as a non-enzymatic antioxidant, mitigating oxidative stress-related damage [176]. Typical dietary sources of vitamin E include nuts and vegetable oils [177]. Lower serum levels of antioxidants, such as vitamin E, are associated with both depression and anxiety [178]. Several studies have shown that antioxidant supplementation can be effective in patients with anxiety and depression by enhancing biological antioxidant defenses [179,180]. However, research on the efficacy of vitamin E supplementation in reducing depressive symptoms is ongoing. Coffee is a valuable source of health-promoting compounds, such as caffeine, which exhibits anti-inflammatory and antioxidant properties similar to GSH, a potent antioxidant protecting cells from free radical damage [181]. In mouse studies, chronic caffeine administration (8 mg/kg/day) increased DA and 5-HT levels, thereby reducing depressive-like behavior [182]. Polyphenols in coffee and tea also provide beneficial effects. Epigallocatechin gallate (EGCG) has demonstrated antidepressant effects in preclinical models by increasing BDNF and 5-HT levels and reducing stress hormones [183,184]. Chlorogenic acid in green tea appears to inhibit monoamine oxidase A, increasing 5-HT levels and exerting antidepressant effects [185]. In mice subjected to exercise and a flavonoid-enriched anti-inflammatory diet, gene expression promoting brain neuroplasticity was increased [186]. Resveratrol, a natural polyphenol found in grapes, berries, and red wine, exhibits antioxidant, anti-inflammatory, and neuroprotective effects [187]. In female mice, resveratrol reduced depressive-like behaviors [188]. Few studies have investigated its effects on human depression, though several assessed mood changes after administration [27,28,29,189,190]. Two studies found no significant differences in mood measures [29,190,191], while one reported reduced anxiety with resveratrol treatment but no changes in depression or other mood aspects [28]. The lack of significant results may relate to lower resveratrol doses relative to body weight compared to doses used in most animal studies. In animal models, resveratrol showed antidepressant effects comparable to several pharmaceutical antidepressants, suggesting its potential as a natural antidepressant [191]. Curcumin, the main active compound in turmeric, is known for its strong anti-inflammatory and antioxidant properties [192]. One meta-analysis suggests that, due to its antioxidant effects, curcumin may alleviate depressive and anxiety symptoms in affected individuals; however, caution is warranted due to small sample sizes [193]. Another study found that curcumin was significantly more effective than placebo in reducing anxiety and depressive symptoms between weeks 4 and 8 of administration. In patients with atypical depression, curcumin showed even greater antidepressant and anxiolytic effects compared to placebo. Although its antidepressant effect was most pronounced between weeks 4 and 8, over the entire treatment period, curcumin was not significantly more effective than placebo in alleviating depressive and anxiety symptoms [30]. In another study, as an adjunct to newly initiated antidepressant treatment (escitalopram or venlafaxine XR), curcumin at 500 mg/day did not enhance treatment efficacy compared to placebo [31]. Curcumin remains a promising compound due to its apparent safety, although further clinical research is needed. 

A study involving 3523 participants from the SU.VI.MAX cohort did not find a clear association between the inflammatory potential of the diet and depression. However, subgroup analyses suggest that a diet with a higher inflammatory potential may increase the risk of developing new depressive symptoms, particularly in men, smokers, and individuals with lower physical activity levels [194]. Recent studies indicate that a 12-week education program on an anti-inflammatory diet for breast cancer patients with depression improved depressive symptoms, increased the anti-inflammatory potential of the diet (reduced E-DII), and partially decreased levels of inflammatory markers TNF-α and CRP [25]. These results suggest that an anti-inflammatory diet may be an effective, low-cost adjunct strategy for managing depression in this specific clinical context. Further research shows that a pro-inflammatory diet, as measured by the DII, is associated with a higher risk of depression and anxiety disorders, as well as increased severity of depressive and anxiety symptoms. This relationship is partially mediated by inflammatory markers and metabolites related to glucose, protein, and lipid metabolism, suggesting that diet influences mental health through both inflammatory and metabolic mechanisms. The findings indicate that modifying the diet toward an anti-inflammatory profile may represent a potential preventive and supportive strategy in treating depressive and anxiety disorders, regardless of sex, body mass index, or socio-economic status [195]. A prospective cohort study based on UK Biobank data showed that adherence to an anti-inflammatory diet, characterized by a low E-DII score, was associated with a reduced risk of depression, whereas a pro-inflammatory diet with a high E-DII increased the likelihood of developing this disorder. These conclusions were consistent across different age groups, BMI ranges, and in individuals with or without hypertension, with certain subpopulations-such as women and less-educated individuals-demonstrating greater sensitivity to the pro-inflammatory potential of the diet [196]. In the Australian Longitudinal Study on Women’s Health, women in the highest pro-inflammatory dietary group were more likely to have lower levels of education, be obese, engage in lower levels of physical activity, and be current smokers-indicating an overall less healthy lifestyle profile [197]. Findings from various studies are inconsistent; some compounds, such as resveratrol and curcumin, show effects only in preclinical models or at specific doses. Further research is necessary to fully elucidate underlying mechanisms, particularly for vitamin D3, and to determine appropriate therapeutic dosages. Well-designed clinical trials are needed to confirm the efficacy of anti-inflammatory dietary interventions and establish optimal therapeutic dosages. Future studies should also explore the long-term effects of anti-inflammatory diets on depressive and anxiety disorders across diverse populations. Additionally, research is required to clarify the interactions between dietary components, individual genetic factors, and lifestyle variables in modulating mental health outcomes.

## 10. Western Diet

The Western diet is a widely used term encompassing several specific dietary patterns. It is typical among residents of medium- and high-income countries and is characterized by a high intake of highly processed foods. This diet is rich in simple sugars, saturated fats, and animal-derived proteins, while providing insufficient amounts of vitamins, fiber, and minerals [198]. Such a dietary pattern contributes to nutritional deficiencies and the development of chronic diseases [199,200]. Practically, it is associated with low consumption of vegetables, fruits, whole grains, and legumes, along with excessive intake of fried foods, sweets, salty snacks, fatty red meat, dairy, refined flour products, and sugar-sweetened beverages [201]. Each of these components has been independently linked to mood disorders and cognitive decline. These foods are typically high in sodium and calories [202]. High consumption of saturated and trans fats disrupts lipid metabolism and adversely affects nervous system function [170]. Diets high in fat also promote weight gain, reduce self-esteem, and worsen mood-factors associated with an increased incidence of depression and neurological disorders [18]. A critical issue is the disrupted ratio of n-6 to n-3 PUFAs. N-6 PUFAs, abundant in high-fat and processed food patterns, significantly exceed the often-deficient n-3 PUFAs. This imbalance exacerbates negative effects on the nervous system and contributes to depression development [203]. Excessive saturated fat intake also impairs physiological balance by reducing hippocampal volume, compromising memory, concentration, and psychomotor performance, and increasing susceptibility to depression [204]. One mechanism linking diet and depression involves elevated pro-inflammatory cytokine production from adipose tissue in response to high-fat intake [50]. Fat excess further increases oxidative stress, damages neurons, and impairs synaptic plasticity. In response, autophagy is activated to maintain homeostasis by regulating lipid metabolism, removing damaged proteins and organelles, and protecting neurons. A key component of this process is the mTOR signaling pathway, which is suppressed via diet-induced phosphorylation, leading to impaired autophagy, increased inflammation, metabolic disturbances, and elevated symptoms of anxiety and depression [205]. Adherence to a Western dietary pattern is also associated with a more sedentary lifestyle, higher consumption of alcohol and tobacco, poor sleep hygiene, and increased social withdrawal-all established contributors to the development and exacerbation of depressive symptoms [206,207]. The Western diet promotes gut dysbiosis by increasing the abundance of *Clostridium*, *Klebsiella*, and *Bilophila wadsworthia*, while reducing *Bifidobacterium* and *Roseburia*. This shift in microbial composition decreases SCFAs production, compromises gut barrier integrity, and heightens inflammatory signaling, which are strongly linked to the pathophysiology of depression [208]. In rodent models, high-fat diets increased the Firmicutes-to-Bacteroidetes ratio, reduced exploratory behavior, and intensified anxiety and memory impairments [209,210]. Other preclinical studies demonstrated that high-calorie diets representing Western-style eating patterns increased the abundance of bacteria from the Clostridiales, Ruminococcaceae, and Bacteroidales groups, correlating with poorer cognitive flexibility, object recognition, and social interaction [211,212]. Prebiotic supplementation reversed stress-induced gut microbiota changes, prevented declines in beneficial bacteria (e.g., *Bifidobacterium*, *Lactobacillus*), and normalized cytokine levels and depressive behaviors in mice [213]. Furthermore, studies confirm the association between sugar-rich diets and depression. High carbohydrate intake increases the risk of diabetes, which may trigger depressive symptoms [214]. Consumption of sugar-sweetened beverages is also correlated with a higher risk of depression, potentially due to several biological mechanisms. These beverages contain high amounts of fructose. In animal studies, rats exposed to high-fructose diets during adolescence exhibited increased anxiety- and depression-like behaviors in adulthood, along with hyperactivation of the HPA axis and elevated glucocorticoid levels. Excessive consumption of these beverages also contributes to obesity, which itself may increase depression risk by impairing HPA axis function [215].

## 11. Ketogenic Diet and Depression

The ketogenic diet (KD) is characterized by very low carbohydrate content, high fat intake, and a moderate protein amount tailored to individual needs [216]. Under conditions of restricted carbohydrate availability, the body undergoes metabolic adaptation, shifting to fatty acids as the primary energy source. Low blood glucose levels reduce insulin, which, together with elevated adrenaline, stimulates hormone-sensitive lipase activity [217]. Consequently, free fatty acids (FFAs) and glycerol are released from adipocytes. FFAs are transported to liver mitochondria, where they undergo β-oxidation to form acetyl-CoA. Under normal conditions, acetyl-CoA enters the Krebs cycle by combining with oxaloacetate. During carbohydrate restriction, however, oxaloacetate is diverted to gluconeogenesis, leaving acetyl-CoA to be converted into ketone bodies: β-hydroxybutyrate, acetoacetate, and acetone [218]. This increase in ketone production is termed nutritional ketosis [219]. Ketone synthesis also rises during fasting, starvation, intense physical activity, and the neonatal period [216]. Ketone bodies serve as an alternative energy source alongside glucose. Studies indicate that during KD, skeletal muscle, heart, and brown adipose tissue rely primarily on β-hydroxybutyrate, whereas the liver, kidneys, and CNS continue to utilize glucose as the main energy source [220].

Emerging research suggests that KD may support therapies for psychiatric disorders. The diet is associated with an increased GABA-to-glutamate ratio. As less glutamate is converted to aspartate, more becomes available for GABA synthesis via glutamate decarboxylase. KD may therefore benefit depression and anxiety disorders, which are often associated with reduced GABA levels. Positive allosteric modulators of GABA, such as benzodiazepines, are commonly used to alleviate depression symptoms, rapidly reducing anxiety and sleep disturbances. KD may complement monoaminergic antidepressants by enhancing CNS GABA levels [221]. Studies suggest that KD can stabilize mood by modulating neurotransmitter regulation, glutamatergic transmission, mitochondrial function, and oxidative stress [222,223]. KD may also improve cognitive functions, including working memory and processing speed [224]. However, some studies indicate that while KD may improve mood, it can also lead to sleep disturbances due to carbohydrate restriction. As sleep quality strongly predicts mental health outcomes, these disturbances may moderate the overall effect of KD on depressive symptoms [225]. Evidence also shows that KD can positively influence gut microbiota across age groups [226]. KD alters the gut microbiota by reducing *Bifidobacterium* and increasing *Akkermansia muciniphila* and *Desulfovibrio*. These changes may reduce gut inflammation and enhance GABAergic signaling in the brain. Prolonged KD, however, may decrease microbial diversity and SCFAs production. Antidepressant effects observed in some patients may result from reduced inflammation and altered neurotransmitter metabolism mediated by the microbiota [227]. Preclinical studies support these findings. Rodent experiments using the Porsolt test demonstrated that KD increases locomotor activity and reduces anxiety- and depression-like behaviors [228,229]. Ketone supplementation in rats also reduced anxiety-like behaviors, suggesting a potential anxiolytic and antidepressant mechanism [230]. In CD-1 mice exposed to KD during the first 30 days of fetal life and fed a standard diet postnatally, adult offspring displayed reduced anxiety and depression-like behaviors, increased physical activity, larger cerebellums, and smaller hypothalamus and corpus callosum volumes compared to controls [231]. Clinical studies are increasingly investigating KD in depression. A recent case series reported that three adults with depression and anxiety disorders who followed a personalized animal-based KD (fat-to-protein+carbohydrate ratio of 1.5:1) for 12–16 weeks showed significant psychological improvements. Two participants achieved full remission within 7 weeks, and the third within 12 weeks, along with improvements in mood, self-compassion, and metabolic health [232]. Ongoing research is exploring ketogenic metabolic therapy (KMT) for treatment-resistant depression (TRD). A 2024 retrospective case study reported a 53-year-old woman with TRD in Bipolar II disorder who underwent KMT, starting with a 1:1 macronutrient ratio, later adjusted to 1.5:1. Blood glucose and ketone levels were monitored alongside mood assessments using the Generalized Anxiety Disorder 7-item Scale (GAD-7), Depression Anxiety Stress Scales (DASS), and PTSD Checklist for DSM-5 (PCL-5). The patient exhibited improved mood stabilization, reduced anxiety, stress, and depressive symptoms, and enhanced quality of life, with effects persisting up to five months post-intervention [233]. A 2025 retrospective case study reported a 47-year-old woman with chronic TRD who achieved complete remission of depressive symptoms within 8 weeks via a ketogenic diet (1.5:1 macronutrient ratio) under multidisciplinary supervision [234]. Additionally, a cohort study by Garner et al. investigated the impact of KD on mental health outcomes. Participants adhering to KD for an average of 23.56 ± 25.7 months reported lower anxiety levels and improved alertness, satisfaction, and calmness using the Perceived Stress Scale (PSS-10) and the Bond-Lader Visual Analog Scales (BL-VAS). In a second group, participants maintaining KD for approximately 44.02 ± 64.97 months showed reduced anxiety and emotional stress, decreased depressive symptoms, and diminished loneliness assessed by the DASS-21 and a brief Three-item Loneliness Scale [235].

## 12. Vegetarian Diet and Depression

A vegetarian diet primarily consists of plant-based products [236]. This type of diet can be categorized into several subtypes depending on the excluded foods. These include the vegan diet, which excludes all animal-derived products; the lacto-ovo-vegetarian diet, which may include eggs and dairy but excludes meat; the pesco-vegetarian diet, which includes fish and limits consumption of other meats to less than once per month; and the semi-vegetarian diet, in which meat and fish are consumed occasionally [237]. In general, adherence to a well-planned vegetarian diet is associated with numerous health benefits and higher dietary quality [238,239,240]. Such diets are typically rich in magnesium and antioxidants, which influence inflammatory markers [241] and neurotransmitter synthesis [242]. However, vegetarian diets may lead to deficiencies in key nutrients, such as iron, vitamin B12, and n-3 PUFAs [243,244,245], which can impair CNS function [246,247]. Plant-based diets enhance microbial diversity and promote the growth of fiber-fermenting bacteria, increasing SCFAs production. High intake of polyphenols and fiber supports *Prevotella* and *Lactobacillus*, which are associated with anti-inflammatory effects. Strict vegan diets, however, may reduce certain microbial species (e.g., *Bacteroides*) and require careful planning to avoid micronutrient deficiencies that might indirectly affect mood [248]. Research findings regarding depression and vegetarian diets are mixed. Semi- and pesco-vegetarians, who consume fish and poultry in addition to other lacto-ovo-vegetarian foods, have shown a higher risk of depression, whereas no significant association has been observed among lacto-ovo-vegetarians [249]. In a study of 90,380 participants from the Constances cohort, vegetarians and pesco-vegetarians exhibited higher rates of depressive symptoms compared to meat-eaters after adjusting for education, occupational status, and income. Specifically, vegetarians with lower legume intake showed a higher risk of depression, suggesting that overall dietary quality may modulate this relationship [250]. In an older Chinese population, a plant-based diet was independently associated with a higher risk of depressive symptoms among men, while no such association was observed in women. This may result from deficiencies in key nutrients such as n-3 PUFAs, vitamin B12, and folate, which affect brain function, as well as from low dietary quality and limited diversity, often linked to lower socioeconomic status [251]. The ALSPAC study demonstrated that in adult men, a vegetarian diet was associated with a higher risk of depressive symptoms, potentially due to the aforementioned nutrient deficiencies. The authors noted the possibility of reverse causation, suggesting that individuals already experiencing depressive symptoms may be more likely to adopt a vegetarian diet due to changes in appetite, food preferences, or efforts to manage health or weight. Ethical or cultural motivations may also correlate with personality traits that influence mood [252]. A meta-analysis reported that vegetarians had statistically higher depression scores than individuals following a traditional diet. However, significant heterogeneity among studies indicates the presence of moderating variables. The authors emphasized that these findings do not allow causal inferences, leaving unclear whether vegetarianism increases depression risk or if individuals with depression are more likely to adopt a vegetarian diet [253]. Michalak et al. observed that in many cases, the initiation of a vegetarian diet occurs after the onset of mood disorders. Two explanations have been proposed: individuals with depression may perceive a vegetarian diet as healthier or develop increased sensitivity to animal suffering, leading to avoidance of animal products [254]. Conversely, some studies indicate an inverse relationship between vegetarian diet adherence and depression [255,256]. In the MASALA study, 13% of participants had depression, with a higher prevalence among non-vegetarians (14.4%) than vegetarians. After adjusting for confounders, vegetarians had 43% lower odds of developing depression. Higher education and income were also associated with lower depression risk, whereas smoking increased risk. The high proportion of vegetarians among South Asians (38% in the cohort) reflects cultural and religious traditions, potentially providing stronger social support and reducing stigma associated with vegetarianism [255]. Unlike vegetarians in Western societies, for this group, vegetarianism is a social and cultural norm, which may explain the inverse association compared to studies in the USA and Europe. Studies of the Seventh-day Adventist (SDA) community in the USA found that vegetarians had lower intakes of EPA, DHA, and AA but higher intakes of ALA and LA compared to meat-eaters. Despite this, vegetarians reported better well-being, particularly among women, who exhibited significantly better mood than meat-eating women. However, limitations included the specific nature of the study population and the absence of measurements of fatty acid concentrations and inflammatory markers in blood [256]. Improved well-being may result from high ALA and LA intake, low AA, and high antioxidant content, suggesting beneficial effects of plant-based diets on mental health. Research on vegetarian diets faces several limitations, including the underlying reasons for adopting the diet, socio-cultural factors, and the diversity of diet types. One study on psychosocial aspects found that most individuals on a meat-free diet cited ethical and environmental reasons (86.9%), while 32.1% believed that a plant-based diet positively affects health, and 42.9% indicated health maintenance as important. Additionally, 64.3% reported aversion to the taste of meat or animal products; none cited fashion or trends as a reason [257]. Individuals adhering to plant-based diets often exhibit higher health-conscious behaviors, including greater physical activity, lower rates of smoking and alcohol use, and stronger ethical motivations, which may contribute to a reduced risk of depression [258,259]. Discrepancies among findings may result from differences in dietary subtypes, nutrient supplementation, duration of adherence, socio-economic status, lifestyle factors, or cultural influences. For example, the protective effect observed in Taiwanese vegetarians [260] may reflect high dietary quality and adequate nutrient intake, whereas higher rates of depressive symptoms in Austrian vegetarians [261] may be influenced by diet motivation, coexisting lifestyle factors, or potential nutrient deficiencies. Further studies are required to clarify the causal relationship between vegetarian diets and depression, considering nutrient adequacy, dietary quality, and dietary subtypes. Future research should explore how socio-cultural factors, lifestyle, and ethical motivations interact with dietary patterns to influence mental health. Additionally, long-term prospective studies and mechanistic research on nutrient-mood pathways are necessary to better understand these associations across diverse populations.

## 13. Conclusions

Depression is a multifactorial mental disorder involving a complex interplay of biological, environmental, and lifestyle factors. This review has highlighted multiple mechanisms implicated in its development, including monoamine imbalance, oxidative and inflammatory processes, HPA axis dysregulation, mitochondrial dysfunction, and alterations in gut microbiota. Evidence suggests that diet may play a supportive role in the management of depression. Specifically, Mediterranean and anti-inflammatory dietary patterns appear most consistently beneficial, likely through modulation of inflammation, improvement of gut microbiota composition, reduction of oxidative stress, and promotion of neuroplasticity. Some studies indicate potential positive effects of the ketogenic diet, although findings remain limited and heterogeneous. Conversely, Western-style diets rich in processed foods and sugars may exacerbate depressive symptoms. Research on vegetarian diets remains inconclusive; some studies report beneficial effects, while others highlight the potential risk of nutritional deficiencies. These discrepancies underscore the need for cautious interpretation and the challenge of translating dietary interventions into clinical practice. Overall, nutritional strategies show promise as adjunctive treatments for depression, yet current evidence is still emerging and must be critically appraised considering methodological limitations and population diversity. From a clinical perspective, integrating anti-inflammatory and balanced dietary approaches may enhance the effectiveness of standard treatments, including pharmacotherapy and psychotherapy. Future research should focus on well-designed randomized controlled trials to clarify the effects of specific nutrients and dietary patterns, identify optimal intervention strategies, and evaluate long-term outcomes. Incorporating dietary considerations into mental health care holds potential to improve patient quality of life, but requires careful, evidence-based implementation.

## Figures and Tables

**Table 1:** Inclusion and exlusion criteria.

Inclusion Criteria	Exlusion Criteria
1. Randomized controlled trials (RCTs) conducted in adult populations, assessing dietary or supplementation interventions. 2. Studies reporting outcomes related to depressive symptoms or mood, measured with validated scales (in clinical depression or other populations). 3. Studies providing a reliable description of results. 4. Meta-analyses. 5. Review articles. 6. Studies published in Polish or English	1. Studies not reporting outcomes related to mood or depression. 2. Studies conducted in pediatric populations. 3. Studies without a control or placebo group. 4. Studies published in languages other than English or Polish

## References

[1] Depression. https://www.who.int/news-room/fact-sheets/detail/depression.

[2] Depresja—Więcej niż Smutek—Środa z Profilaktyką w OW NFZ. https://www.nfz.gov.pl/aktualnosci/aktualnosci-oddzialow/depresja-wiecej-niz-smutek-sroda-z-profilaktyka-w-ow-nfz,590.html.

[3] De Zwart P.L., Jeronimus B.F., de Jonge P. (2019). Empirical evidence for definitions of episode, remission, recovery, relapse and recurrence in depression: A systematic review. Epidemiol. Psychiatr. Sci..

[4] Ross B.M. (2007). ω−3 Fatty acid deficiency in major depressive disorder is caused by the interaction between diet and a genetically determined abnormality in phospholipid metabolism. Med. Hypotheses.

[5] Bujnowska-Fedak M., Grata-Borkowska U., Sapilak B. (2012). Otępienie i depresja u pacjentów w podeszłym wieku w Praktyce Lekarza Rodzinnego. Fam. Med. Prim. Care Rev..

[6] American Psychiatric Association (2013). Diagnostic and Statistical Manual of Mental Disorders.

[7] Brown S.-L., Bleich A., Van Praag H.M. (2023). The monoamine hypothesis of depression: The case for serotonin. Role of Serotonin in Psychiatric Disorders.

[8] Capuco A., Urits I., Hasoon J., Chun R., Gerald B., Wang J.K., Kassem H., Ngo A.L., Abd-Elsayed A., Simopoulos T. (2020). Current perspectives on gut microbiome dysbiosis and depression. Adv. Ther..

[9] Mikulska J., Juszczyk G., Gawrońska-Grzywacz M., Herbet M. (2021). HPA axis in the pathomechanism of depression and schizophrenia: New therapeutic strategies based on its participation. Brain Sci..

[10] Liu T., Zhong S., Liao X., Chen J., He T., Lai S., Jia Y. (2015). A meta-analysis of oxidative stress markers in depression. PLoS ONE.

[11] Schiepers O.J., Wichers M.C., Maes M. (2005). Cytokines and major depression. Prog. Neuro-Psychopharmacol. Biol. Psychiatry.

[12] Allen J., Romay-Tallon R., Brymer K.J., Caruncho H.J., Kalynchuk L.E. (2018). Mitochondria and mood: Mitochondrial dysfunction as a key player in the manifestation of depression. Front. Neurosci..

[13] Cruwys T., Haslam S.A., Dingle G.A., Haslam C., Jetten J. (2014). Depression and social identity: An integrative review. Pers. Soc. Psychol. Rev..

[14] Joormann J., Stanton C.H. (2016). Examining emotion regulation in depression: A review and future directions. Behav. Res. Ther..

[15] Martinsen E.W. (2008). Physical activity in the prevention and treatment of anxiety and depression. Nord. J. Psychiatry.

[16] Samochowiec J., Dudek D., Mazur J.K., Murawiec S., Rymaszewska J., Cubała W., Heitzman J., Szulc A., Bała M., Gałecki P. (2021). Leczenie farmakologiczne epizodu depresji i zaburzeń depresyjnych nawracających—Wytyczne Polskiego Towarzystwa Psychiatrycznego i Konsultanta Krajowego ds. Psychiatrii Dorosłych. Psychiatr. Pol..

[17] Popiel A., Pragłowska E. (2009). Psychoterapia poznawczo-behawioralna—Praktyka oparta na badaniach empirycznych. Psychiatr. Prak. Klin..

[18] Majkutewicz P., Tyszko P., Okręglicka K. (2014). Leczenie żywieniowe depresji. Fam. Med. Prim. Care Rev..

[19] Jacka F.N., O’Neil A., Opie R., Itsiopoulos C., Cotton S., Mohebbi M., Castle D., Dash S., Mihalopoulos C., Chatterton M.L. (2017). A randomised controlled trial of dietary improvement for adults with major depression (the ‘SMILES’ trial). BMC Med..

[20] Siwek M., Dudek D., Schlegel-Zawadzka M., Morawska A., Piekoszewski W., Opoka W., Zięba A., Pilc A., Popik P., Nowak G. (2010). Serum zinc level in depressed patients during zinc supplementation of imipramine treatment. J. Affect. Disord..

[21] Benton D., Cook R. (1991). The impact of selenium supplementation on mood. Biol. Psychiatry.

[22] Mehrpooya M., Hajiaghaee R., Jahani F., Mohammadi M.R., Tabrizi M., Sadr S.S. (2018). Evaluating the effect of coenzyme Q10 augmentation on treatment of bipolar depression: A double-blind controlled clinical trial. J. Clin. Psychopharmacol..

[23] Harbottle L., Schonfelder N. (2008). Nutrition and depression: A review of the evidence. J. Ment. Health.

[24] Chang J.P., Chang S.S., Yang H.T., Chen H.T., Chien Y.C., Yang B., Su H., Su K.P. (2020). Omega-3 polyunsaturated fatty acids in cardiovascular diseases comorbid major depressive disorder—Results from a randomized controlled trial. Brain Behav. Immun..

[25] Cheng L., Chen Y., He J., Cheng X., Wang Y., Lin X., Huang Z., Miao X., Xia S. (2025). Effects of 12-Week Anti-Inflammatory Dietary Education on Depressive Symptoms Among Depressed Patients with Breast Cancer Undergoing Adjuvant Chemotherapy: A Randomized Controlled Trial. Nutrients.

[26] Kaviani M., Ghoreishi A., Rezaei F., Moloodi R., Akbari H., Salami M. (2020). Effects of vitamin D supplementation on depression and some involved neurotransmitters. J. Affect. Disord..

[27] Davinelli S., Scapagnini G., Marzatico F., Nobile V., Ferrara N., Corbi G. (2017). Influence of equol and resveratrol supplementation on health-related quality of life in menopausal women: A randomized, placebo-controlled study. Maturitas.

[28] Evans H.M., Howe P.R., Wong R.H. (2017). Effects of resveratrol on cognitive performance, mood and cerebrovascular function in post-menopausal women; a 14-week randomised placebo-controlled intervention trial. Nutrients.

[29] Wightman E.L., Reay J.L., Haskell C.F., Williamson G., Dew T.P., Kennedy D.O. (2014). Effects of resveratrol alone or in combination with piperine on cerebral blood flow parameters and cognitive performance in human subjects: A randomised, double-blind, placebo-controlled, cross-over investigation. Br. J. Nutr..

[30] Lopresti A.L., Maes M., Maker G.L., Hood S.D., Drummond P.D. (2014). Curcumin for the treatment of major depression: A randomised, double-blind, placebo controlled study. J. Affect. Disord..

[31] Bergman J., Miodownik C., Bersudsky Y., Sokolik S., Lerner P.P., Kreinin A., Polakiewicz J., Lerner V. (2013). Curcumin as an add-on to antidepressive treatment: A randomized, double-blind, placebo-controlled, pilot clinical study. Clin. Neuropharmacol..

[32] Hamon M., Blier P. (2013). Monoamine neurocircuitry in depression and strategies for new treatments. Prog. Neuro-Psychopharmacol. Biol. Psychiatry.

[33] Descarries L., Watkins K.C., Garcia S., Beaudet A. (1982). The serotonin neurons in nucleus raphe dorsalis of adult rat: A light and electron microscope radioautographic study. J. Comp. Neurol..

[34] Benkelfat C., Ellenbogen M.A., Dean P., Palmour R.M., Young S.N. (1994). Mood-lowering effect of tryptophan depletion. Enhanced susceptibility in young men at genetic risk for major affective disorders. Arch. Gen. Psychiatry.

[35] Delgado P.L., Charney D.S., Price L.H., Aghajanian G.K., Landis H., Heninger G.R. (1990). Serotonin function and the mechanism of antidepressant action. Reversal of antidepressant-induced remission by rapid depletion of plasma tryptophan. Arch. Gen. Psychiatry.

[36] Wolkowitz O.M., Epel E.S., Reus V.I. (2001). Stress hormone-related psychopathology: Pathophysiological and treatment implications. World J. Biol. Psychiatry.

[37] Roy A., De Jong J., Linnoila M. (1989). Cerebrospinal fluid monoamine metabolites and suicidal behavior in depressed patients. A 5-year follow-up study. Arch. Gen. Psychiatry.

[38] Richelson E. (2001). Pharmacology of antidepressants. Mayo Clin. Proc..

[39] Leonard B.E. (2001). Stress, norepinephrine and depression. J. Psychiatry Neurosci..

[40] Ordway G.A., Smith K.S., Haycock J.W. (1994). Elevated tyrosine hydroxylase in the locus coeruleus of suicide victims. J. Neurochem..

[41] Klimek V., Stockmeier C., Overholser J., Meltzer H.Y., Kalka S., Dilley G., Ordway G.A. (1997). Reduced levels of norepinephrine transporters in the locus coeruleus in major depression. J. Neurosci..

[42] Coppen A. (1967). The biochemistry of affective disorders. Br. J. Psychiatry.

[43] Randrup A., Braestrup C. (1977). Uptake inhibition of biogenic amines by newer antidepressant drugs: Relevance to the dopamine hypothesis of depression. Psychopharmacology.

[44] Nestler E.J., Carlezon W.A. (2006). The mesolimbic dopamine reward circuit in depression. Biol. Psychiatry.

[45] Jokinen J., Nordström A.L., Nordström P. (2007). The relationship between CSF HVA/5-HIAA ratio and suicide intent in suicide attempters. Arch. Suicide Res..

[46] Pan J.X., Xia J.J., Deng F.L., Liang W.W., Wu J., Yin B.M., Dong M.X., Chen J.J., Ye F., Wang H.Y. (2018). Diagnosis of major depressive disorder based on changes in multiple plasma neurotransmitters: A targeted metabolomics study. Transl. Psychiatry.

[47] Fogaça M.V., Duman R.S. (2019). Cortical GABAergic dysfunction in stress and depression: New insights for therapeutic interventions. Front. Cell. Neurosci..

[48] Hersey M., Hashemi P., Reagan L.P. (2022). Integrating the monoamine and cytokine hypotheses of depression: Is histamine the missing link?. Eur. J. Neurosci..

[49] Jauhar S., Cowen P.J., Browning M. (2023). Fifty years on: Serotonin and depression. J. Psychopharmacol..

[50] Miller A.H., Raison C.L. (2016). The role of inflammation in depression: From evolutionary imperative to modern treatment target. Nat. Rev. Immunol..

[51] Pariante C.M., Lightman S.L. (2008). The HPA axis in major depression: Classical theories and new developments. Trends Neurosci..

[52] Park H.Y., Suh C.H., Heo H., Shim W.H., Kim S.J. (2022). Diagnostic performance of hippocampal volumetry in Alzheimer's disease or mild cognitive impairment: A meta-analysis. Eur. Radiol..

[53] Xu H.W., Li X.C., Li H.D., Ruan H.Z., Liu Z.Z. (2000). Effects of corticotrophin on pain behavior and BDNF, CRF levels in frontal cortex of rats suffering from chronic pain. Acta Pharmacol. Sin..

[54] Farooqi N.A.I., Scotti M., Lew J.M., Botteron K.N., Karama S., McCracken J.T., Nguyen T.V. (2018). Role of DHEA and cortisol in prefrontal-amygdalar development and working memory. Psychoneuroendocrinology.

[55] Bhatt S., Nagappa A.N., Patil C.R. (2020). Role of oxidative stress in depression. Drug Discov. Today.

[56] Correia A.S., Cardoso A., Vale N. (2023). Oxidative Stress in Depression: The Link with the Stress Response, Neuroinflammation, Serotonin, Neurogenesis and Synaptic Plasticity. Antioxidants.

[57] Jimenez-Fernandez S., Tuck S.J., Morris C., Murray C., McAllister-Williams R.H. (2015). Oxidative stress and antioxidant parameters in patients with major depressive disorder compared to healthy controls before and after antidepressant treatment: Results from a meta-analysis. J. Clin. Psychiatry.

[58] Irie M., Hirabayashi Y., Suzuki K., Suzuki M., Tanaka M., Kato N. (2003). Depressive state relates to female oxidative DNA damage via neutrophil activation. Biochem. Biophys. Res. Commun..

[59] Gałecka E., Szemraj J., Florkowski A., Bieńkowski P., Bieńkowski P., Gałecki P. (2008). Wybrane substancje nieenzymatyczne uczestniczące w procesie obrony przed nadmiernym wytwarzaniem wolnych rodników. Pol. Merk. Lek..

[60] Halliwell B. (2006). Oxidative stress and neurodegeneration: Where are we now?. J. Neurochem..

[61] Miller E., Rutkowski M. (2006). Udział i rola ważniejszych czynników biochemicznych w udarze niedokrwiennym mózgu. Pol. Merk. Lek..

[62] Sakharov D., Elstak E., Chernyak B., Wirtz K. (2005). Prolonged lipid oxidation after photodynamic treatment. Study with oxidation-sensitive probe C11-BODIPY^581/591^. FEBS Lett..

[63] Ponczek M.B., Wachowicz B. (2005). Oddziaływanie reaktywnych form tlenu i azotu z białkami. Postępy Biochem..

[64] Bailey S.M., Landar A., Darley-Usmar V. (2005). Mitochondrial proteomics in free radical research. Free Radic. Biol. Med..

[65] Wigner P., Śliwiński T. (2022). Rola stresu oksydacyjnego i nitracyjnego oraz szlaku katabolitów tryptofanu w patogenezie depresji. Postępy Biochem..

[66] Stefanescu C., Ciobica A. (2012). The relevance of oxidative stress status in first episode and recurrent depression. J. Affect. Disord..

[67] Yager S., Forlenza M.J., Miller G.E. (2010). Depression and oxidative damage to lipids. Psychoneuroendocrinology.

[68] Forlenza M.J., Miller G.E. (2006). Increased serum levels of 8-hydroxy-2′-deoxyguanosine in clinical depression. Psychosom. Med..

[69] Chung C.P., Wang S.Y., Wu C.S., Chang H.A., Huang T.L. (2013). Increased oxidative stress in patients with depression and its relationship to treatment. Psychiatry Res..

[70] Herken H., Gurel A., Selek S., Armutcu F., Bulut M., Altindag A. (2007). Adenosine deaminase, nitric oxide, superoxide dismutase, and xanthine oxidase in patients with major depression: Impact of antidepressant treatment. Arch. Med. Res..

[71] Michel T.M., Riederer P., Klammer N., Ackenheil M., Maier W., Scherk H. (2010). Increased xanthine oxidase in the thalamus and putamen in depression. World J. Biol. Psychiatry.

[72] Marreiro D.N., Cruz K.J., Morais J.B.S., Beserra J.B., Severo J.S., de Oliveira A.R.S. (2017). Zinc and oxidative stress: Current mechanisms. Antioxidants.

[73] Swardfager W., Herrmann N., McIntyre R.S., Mazereeuw G., Goldberger K., Harimoto T., Lanctôt K.L. (2013). Zinc in depression: A meta-analysis. Biol. Psychiatry.

[74] Maes M., Declerck L., Scharpé S., Bosmans E., Stevens W., Kenis G., De Mayer E., Neels H. (1994). Hypozincemia in depression. J. Affect. Disord..

[75] Grieger J.A., Nowson C.A., Ackland L.M. (2009). Nutritional and functional status indicators in residents of a long-term care facility. J. Nutr. Elder..

[76] Amani R., Saeidi S., Nazari Z., Nematpour S. (2010). Correlation between dietary zinc intakes and its serum levels with depression scales in young female students. Biol. Trace Elem. Res..

[77] Nowak G., Zieba A., Dudek D., Krosniak M., Szymaczek M., Schlegel-Zawadzka M. (1999). Serum trace elements in animal models and human depression. Part I. Zinc. Hum. Psychopharmacol..

[78] Moylan S., Maes M., Wray N.R., Berk M. (2012). The neuroprogressive nature of major depressive disorder: Pathways to disease evolution and resistance, and therapeutic implications. Mol. Psychiatry.

[79] Swardfager W., Herrmann N., McIntyre R.S., Mazereeuw G., Goldberger K., Cha D.S., Schwartz Y., Lanctôt K.L. (2013). Potential roles of zinc in the pathophysiology and treatment of major depressive disorder. Neurosci. Biobehav. Rev..

[80] Leonard B., Maes M. (2012). Mechanistic explanations how cell-mediated immune activation, inflammation and oxidative and nitrosative stress pathways and their sequels and concomitants play a role in the pathophysiology of unipolar depression. Neurosci. Biobehav. Rev..

[81] Bjørklund G., Shanaida M., Lysiuk R., Antonyak H., Klishch I., Shanaida V., Peana M. (2022). Selenium: An antioxidant with a critical role in anti-aging. Molecules.

[82] Benton D. (2002). Selenium intake, mood and other aspects of psychological functioning. Nutr. Neurosci..

[83] Derbeneva S.A., Bogdanov A.R., Pogozheva A.V., Gladyshev O.A., Vasilevskaia L.S., Zorin S.N., Mazo V.K. (2012). Effect of diet enriched with selenium on the psycho-emotional and adaptive capacity of patients with cardiovascular diseases and obesity. Vopr. Pitan..

[84] Finney S.J., DiStefano C., Hancock G.R., Mueller R.O. (2006). Nonnormal and categorical data in structural equation modeling. Structural Equation Modeling: A Second Course.

[85] Browne M.W., Cudeck R., Bollen K.A., Long J.S. (1993). Alternative ways of assessing model fit. Testing Structural Equation Models.

[86] Wang J., Um P., Dickerman B.A., Liu J. (2018). Zinc, magnesium, selenium and depression: A review of the evidence, potential mechanisms and implications. Nutrients.

[87] Mertens K., Lowes D., Webster N., Talib J., Hall L., Davies M.J., Beattie J., Galley H. (2015). Low zinc and selenium concentrations in sepsis are associated with oxidative damage and inflammation. Br. J. Anaesth..

[88] Prystupa A., Kiciński P., Luchowska-Kocot D., Błażewicz A., Niedziałek J., Mizerski G., Jojczuk M., Ochal A., Sak J.J., Załuska W. (2017). Association between serum selenium concentrations and levels of proinflammatory and profibrotic cytokines—Interleukin-6 and growth differentiation factor-15, in patients with alcoholic liver cirrhosis. Int. J. Environ. Res. Public Health.

[89] Duntas L. (2009). Selenium and inflammation: Underlying anti-inflammatory mechanisms. Horm. Metab. Res..

[90] Castaño A., Ayala A., Rodrıguez-Gómez J.A., Herrera A.J., Cano J., Machado A. (1997). Low selenium diet increases the dopamine turnover in prefrontal cortex of the rat. Neurochem. Int..

[91] Santos A.C., Passos A.F.F., Holzbach L.C., Cardoso B.R., Santos M.A., Coelho A.S.G., Cominetti C., Almeida G.M. (2025). Lack of sufficient evidence to support a positive role of selenium status in depression: A systematic review. Nutr. Rev..

[92] Parnham M.J., Sies H. (2013). The early research and development of ebselen. Biochem. Pharmacol..

[93] Ramli F.F., Cowen P.J., Godlewska B.R. (2022). The potential use of ebselen in treatment-resistant depression. Pharmaceuticals.

[94] Adler G. (2009). Cytokiny w początkowych etapach odpowiedzi odpornościowej. Reumatologia.

[95] Margielewska S. (2013). Rola układu odpornościowego w powstawaniu depresji. Wszechświat.

[96] Maes M. (2011). Depression is an inflammatory disease, but cell-mediated immune activation is the key component of depression. Prog. Neuro-Psychopharmacol. Biol. Psychiatry.

[97] Stuart M.J., Baune B.T. (2012). Depression and type 2 diabetes: Inflammatory mechanisms of a psychoneuroendocrine co-morbidity. Neurosci. Biobehav. Rev..

[98] Turnbull A.V., Rivier C. (1995). Regulation of the HPA axis by cytokines. Brain Behav. Immun..

[99] Wachowska K., Gałecki P. (2021). Inflammation and cognition in depression: A narrative review. J. Clin. Med..

[100] Gold P.W. (2015). The organization of the stress system and its dysregulation in depressive illness. Mol. Psychiatry.

[101] Duman R.S., Monteggia L.M. (2006). A neurotrophic model for stress-related mood disorders. Biol. Psychiatry.

[102] Rolfe D.F., Brown G.C. (1997). Cellular energy utilization and molecular origin of standard metabolic rate in mammals. Physiol. Rev..

[103] Wong-Riley M.T.T., Niehoff M.L., Wu C. (1989). Effect of retinal impulse blockage on cytochrome oxidase-rich zones in the macaque striate cortex: I. Quantitative electron-microscopic (EM) analysis of neurons. Vis. Neurosci..

[104] Zhu X.-H., Zhang Y., Tian R., Lei H., Zhang N., Xin L., Lohrenz T., Zhuang Z., Rao H., Chen W. (2012). Quantitative imaging of energy expenditure in human brain. Neuroimage.

[105] Bansal Y., Kuhad A. (2016). Mitochondrial dysfunction in depression. Curr. Neuropharmacol..

[106] Caruso G., Guerriero F., Fusi C., Ghelardini C., Bizzarri M. (2019). The many faces of mitochondrial dysfunction in depression: From pathology to treatment. Front. Pharmacol..

[107] Khan M., Baussan Y., Hebert-Chatelain E. (2023). Connecting dots between mitochondrial dysfunction and depression. Biomolecules.

[108] Couto N., Wood J., Barber J. (2016). The role of glutathione reductase and related enzymes on cellular redox homoeostasis network. Free Radic. Biol. Med..

[109] Tuura R.O., Miller J.L., Scharinger M., Gong Q., Deakin J.F.W., Laufs H., Suckling J., Walter M. (2023). Prefrontal glutathione levels in major depressive disorder are linked to a lack of positive affect. Brain Sci..

[110] Godlewska B.R., Near J., Cowen P.J. (2015). Neurochemistry of major depression: A study using magnetic resonance spectroscopy. Psychopharmacology.

[111] Frączkowski K., Kowalska M., Nowak P., Zieliński T., Wiśniewski J. (2013). Wpływ koenzymu Q10 na stres oksydacyjny komórki indukowany ksenobiotykami. Acta Bio-Opt. Inform. Med. Inżyn. Biomedyczna.

[112] Maes M., Mihaylova I., Rief W., Kubera M., Leunis J.-C. (2009). Lower plasma Coenzyme Q10 in depression: A marker for treatment resistance and chronic fatigue in depression and a risk factor to cardiovascular disorder in that illness. Neuroendocrinol. Lett..

[113] Packer L., Witt E.H., Tritschler H.J. (1995). Alpha-lipoic acid as a biological antioxidant. Free Radic. Biol. Med..

[114] Ostadmohammadi V., Raygan F., Asemi Z. (2022). Alpha-lipoic acid administration affects psychological status and markers of inflammation and oxidative damage in patients with type 2 diabetes and coronary heart disease. J. Diabetes Metab. Disord..

[115] Góralczyk-Bińkowska A., Szmajda-Krygier D., Kozłowska E. (2022). The Microbiota-Gut-Brain Axis in Psychiatric Disorders. Int. J. Mol. Sci..

[116] Lima-Ojeda J.M., Rupprecht R., Baghai T.C. (2020). Darmflora und Depression. Nervenarzt.

[117] Socała K., Doboszewska U., Szopa A., Serefko A., Włodarczyk M., Zielińska A., Poleszak E., Fichna J., Wlaź P. (2021). The role of microbiota-gut-brain axis in neuropsychiatric and neurological disorders. Pharmacol. Res..

[118] Żakowicz J., Bramorska A., Zarzycka W., Kovbasiuk A., Kuć K., Brzezicka A. (2020). Wpływ mikrobioty jelitowej na mózg, funkcje poznawcze i emocje. Kosmos. Probl. Nauk. Biol..

[119] Calabrò S., Kankowski S., Cescon M., Gambarotta G., Raimondo S., Haastert-Talini K., Ronchi G. (2023). Impact of Gut Microbiota on the Peripheral Nervous System in Physiological, Regenerative and Pathological Conditions. Int. J. Mol. Sci..

[120] Halverson T., Alagiakrishnan K. (2020). Gut microbes in neurocognitive and mental health disorders. Ann. Med..

[121] Pedroza Matute S., Iyavoo S. (2023). Exploring the gut microbiota: Lifestyle choices, disease associations, and personal genomics. Front. Nutr..

[122] Singh J., Vanlallawmzuali, Singh A., Biswal S., Zomuansangi R., Lalbiaktluangi C., Singh B.P., Singh P.K., Vellingiri B., Iyer M. (2024). Microbiota-brain axis: Exploring the role of gut microbiota in psychiatric disorders—A comprehensive review. Asian J. Psychiatr..

[123] Młynarska E., Gadzinowska J., Tokarek J., Forycka J., Szuman A., Franczyk B., Rysz J. (2022). The Role of the Microbiome-Brain-Gut Axis in the Pathogenesis of Depressive Disorder. Nutrients.

[124] Anand N., Gorantla V.R., Chidambaram S.B. (2023). The Role of Gut Dysbiosis in the Pathophysiology of Neuropsychiatric Disorders. Cells.

[125] Sonali S., Ray B., Ahmed Tousif H., Rathipriya A.G., Sunanda T., Mahalakshmi A.M., Rungratanawanich W., Essa M.M., Qoronfleh M.W., Chidambaram S.B. (2022). Mechanistic Insights into the Link between Gut Dysbiosis and Major Depression: An Extensive Review. Cells.

[126] Gałecka M., Basińska A.M., Bartnicka A. (2018). Znaczenie mikrobioty jelitowej w kształtowaniu zdrowia człowieka—Implikacje w praktyce lekarza rodzinnego. Forum Med. Rodz..

[127] Rudzki L., Frank M., Szulc A., Gałecka M., Szachta P., Barwinek D. (2012). Od jelit do depresji—Rola zaburzeń ciągłości bariery jelitowej i następcza aktywacja układu immunologicznego w zapalnej hipotezie depresji. Neuropsychiatr. Neuropsychol..

[128] Yadav H., Jaldhi, Bhardwaj R., Anamika, Bakshi A., Gupta S., Maurya S.K. (2023). Unveiling the role of gut-brain axis in regulating neurodegenerative diseases: A comprehensive review. Life Sci..

[129] Kendler K.S., Karkowski L.M., Prescott C.A. (1999). Causal relationship between stressful life events and the onset of major depression. Am. J. Psychiatry.

[130] Pechtel P., Pizzagalli D.A. (2011). Effects of early life stress on cognitive and affective function: An integrated review of human literature. Psychopharmacology.

[131] Willner P. (2005). Chronic mild stress (CMS) revisited: Consistency and behavioural-neurobiological concordance in the effects of CMS. Neuropsychobiology.

[132] Yang W., Li H., Cheng Z., Lu Y., Li W., Feng J., Wang L., Cheng J. (2022). Dex modulates the balance of water-electrolyte metabolism by depressing the expression of AVP in PVN. Front. Pharmacol..

[133] Herselman M.F., Bailey S., Bobrovskaya L. (2022). The Effects of Stress and Diet on the “Brain–Gut” and “Gut–Brain” Pathways in Animal Models of Stress and Depression. Int. J. Mol. Sci..

[134] Stephens M.A.C., Wand G. (2012). Stress and the HPA axis: Role of glucocorticoids in alcohol dependence. Alcohol. Res. Curr. Rev..

[135] Oray M., Abu Samra K., Ebrahimiadib N., Meese H., Foster C.S. (2016). Long-term side effects of glucocorticoids. Expert. Opin. Drug Saf..

[136] De Kloet E.R., Vreugdenhil E., Oitzl M.S., Joëls M. (1998). Brain corticosteroid receptor balance in health and disease. Endocr. Rev..

[137] Harrison P.J. (2002). The neuropathology of primary mood disorder. Brain.

[138] Naranjo C.A., Tremblay L.K., Busto U.E. (2001). The role of the brain reward system in depression. Prog. Neuro-Psychopharmacol. Biol. Psychiatry.

[139] Brown E.S., Woolston D.J., Frol A., Bobadilla L., Khan D.A., Hanczyc M., Rush A.J., Fleckenstein J., Babcock E., Cullum C.M. (2004). Hippocampal volume, spectroscopy, cognition, and mood in patients receiving corticosteroid therapy. Biol. Psychiatry.

[140] Duman R.S., Malberg J., Nakagawa S. (2001). Regulation of adult neurogenesis by psychotropic drugs and stress. J. Pharmacol. Exp. Ther..

[141] Sousa N., Lukoyanov N.V., Madeira M.D., Almeida O.F., Paula-Barbosa M.M. (2000). Reorganization of the morphology of hippocampal neurites and synapses after stress-induced damage correlates with behavioral improvement. Neuroscience.

[142] Keys A., Menotti A., Karvonen M.J., Aravanis C., Blackburn H., Buzina R., Djordjevic B.S., Dontas A.S., Fidanza F., Keys M.H. (1986). The diet and 15-year death rate in the seven countries study. Am. J. Epidemiol..

[143] Guasch-Ferré M., Willett W.C. (2021). The Mediterranean diet and health: A comprehensive overview. J. Intern. Med..

[144] Rudzińska A., Kowalczyk M., Nowak L., Wiśniewska K., Zieliński P. (2008). Czy dieta śródziemnomorska może zmniejszać ryzyko depresji u evidence. J. Ment. Health.

[145] Merra G., Noce A., Marrone G., Cintoni M., Tarsitano M.G., Capacci A., De Lorenzo A. (2020). Influence of Mediterranean Diet on Human Gut Microbiota. Nutrients.

[146] Seremak-Mrozikiewicz A. (2013). Znaczenie metabolizmu folianów w rozwoju powikłań u kobiet ciężarnych. Ginekol. Pol..

[147] Sánchez-Villegas A., Henríquez P., Bes-Rastrollo M., Doreste J. (2006). Mediterranean diet and depression. Public Health Nutr..

[148] Liao Y., Xie B., Zhang H., He Q., Guo L., Subramanieapillai M., Fan B., Lu C., McIntyre R.S. (2019). Efficacy of omega-3 PUFAs in depression: A meta-analysis. Transl. Psychiatry.

[149] Pacheco-Ordaz R., Wall-Medrano A., Goñi M.G., Ramos-Clamont-Montfort G., Ayala-Zavala J.F., González-Aguilar G.A. (2018). Effect of phenolic compounds on the growth of selected probiotic and pathogenic bacteria. Lett. Appl. Microbiol..

[150] Gopinath B., Flood V.M., Kifley A., Louie J.C., Mitchell P. (2016). Association Between Carbohydrate Nutrition and Successful Aging Over 10 Years. J. Gerontol. A Biol. Sci. Med. Sci..

[151] De Oliveira Meller F., Meller Manosso L., Schäfer A.A. (2021). The influence of diet quality on depression among adults and elderly: A population-based study. J. Affect. Disord..

[152] Masana M.F., Garcia R., Lopez J., Fernandez P., Ruiz A., Ortega L. (2018). Mediterranean diet and depression among older individuals: The multinational MEDIS study. Exp. Gerontol..

[153] Psaltopoulou T., Panagiotakos D.B., Sergentanis T.N., Kosti R., Scarmeas N., Anastasiou C.A., Stefanadis C. (2008). Diet, physical activity and cognitive impairment among elders: The EPIC–Greece cohort (European Prospective Investigation into Cancer and Nutrition). Public Health Nutr..

[154] Roberts S.H., Horan M., Lewis S., Anderson I.M. (2007). Folate Augmentation of Treatment–Evaluation for Depression (FolATED): Protocol of a randomised controlled trial. BMC Psychiatry.

[155] Hibbeln J.R. (1998). Fish consumption and major depression. Lancet.

[156] Kate N., Grover S., Agarwal M. (2010). Does B12 deficiency lead to lack of treatment response to conventional antidepressants?. Psychiatry.

[157] Chopra C., Mandalika S., Kinger N. (2021). Does diet play a role in the prevention and management of depression among adolescents? A narrative review. Nutr. Health.

[158] Sánchez-Villegas A., Delgado-Rodríguez M., Alonso A., Schlatter J., Lahortiga F., Serra Majem L., Martínez-González M.A. (2009). Association of the Mediterranean dietary pattern with the incidence of depression: The Seguimiento Universidad de Navarra/University of Navarra follow-up (SUN) cohort. Arch. Gen. Psychiatry.

[159] Li X., Zhang Y., Wang L., Chen H., Liu J., Zhao Q. (2022). Dietary inflammatory potential and the incidence of depression and anxiety: A meta-analysis. J. Health Popul. Nutr..

[160] Tolkien K., Bradburn S., Murgatroyd C. (2019). An anti-inflammatory diet as a potential intervention for depressive disorders: A systematic review and meta-analysis. Clin. Nutr..

[161] Shivappa N., Steck S.E., Hurley T.G., Hussey J.R., Hébert J.R. (2014). Designing and developing a literature-derived, population-based dietary inflammatory index. Public Health Nutr..

[162] Appleton K.M., Rogers P.J., Ness A.R. (2006). Effects of n–3 long-chain polyunsaturated fatty acids on depressed mood: Systematic review of published trials. Am. J. Clin. Nutr..

[163] DiGirolamo A.M., Ramirez-Zea M. (2009). Role of zinc in maternal and child mental health. Am. J. Clin. Nutr..

[164] Ortega M.A., Requejo A.M., Romero-González J., García-Luna P.P., Delgado P., López M.G., Sánchez R.A. (2022). Biological role of nutrients, food and dietary patterns in the prevention and clinical management of major depressive disorder. Nutrients.

[165] Lee A.R.Y.B., Wong C.L., Yau Y.H.C., Chan H.Y., Leung P.S., Chan K.W. (2022). Vitamin E, alpha-tocopherol, and its effects on depression and anxiety: A systematic review and meta-analysis. Nutrients.

[166] Kurowska K., Ślusarczyk J., Nowak G. (2021). Znaczenie witaminy D w zapobieganiu i leczeniu depresji. Med. Ogólna Nauki o Zdrowiu.

[167] Lakhan S.E., Vieira K.F. (2008). Nutritional therapies for mental disorders. Nutr. J..

[168] Martins J.G. (2009). EPA but not DHA appears to be responsible for the efficacy of omega-3 long chain polyunsaturated fatty acid supplementation in depression: Evidence from a meta-analysis of randomized controlled trials. J. Am. Coll. Nutr..

[169] Féart C., Samieri C., Allès B., Barberger-Gateau P. (2008). Plasma eicosapentaenoic acid is inversely associated with severity of depressive symptomatology in the elderly: Data from the Bordeaux sample of the Three-City Study. Am. J. Clin. Nutr..

[170] Tsaluchidu S., Haddad P.M., Carrillo O., Wurtman R.J., Eyles D.W. (2008). Fatty acids and oxidative stress in psychiatric disorders. BMC Psychiatry.

[171] Bakhtiari-Dovvombaygi H., Shabani M., Fathollahi Y., Roghani M., Khodagholi F. (2021). Beneficial effects of vitamin D on anxiety and depression-like behaviors induced by unpredictable chronic mild stress by suppression of brain oxidative stress and neuroinflammation in rats. Naunyn-Schmiedeberg’s Arch. Pharmacol..

[172] Koshkina A., Wessjohann L., Schmitz D., Winter R., Stahr P., Lutz B. (2019). Effects of vitamin D3 in long-term ovariectomized rats subjected to chronic unpredictable mild stress: BDNF, NT-3, and NT-4 implications. Nutrients.

[173] Sedaghat K., Vahid-Ansari F., Albert P.R. (2019). Mesolimbic dopamine system and its modulation by vitamin D in a chronic mild stress model of depression in the rat. Behav. Brain Res..

[174] Wacker M., Holick M.F. (2013). Sunlight and Vitamin D: A global perspective for health. Dermatoendocrinology.

[175] Cui C., Song Y., Li Y., Zhang J., Wang R., Zhang Y., Zhang Y., Wang X. (2018). Vitamin D Receptor Activation Influences NADPH Oxidase (NOX_2_) Activity and Protects against Neurological Deficits and Apoptosis in a Rat Model of Traumatic Brain Injury. Oxid. Med. Cell. Longev..

[176] Zielińska A., Nowak I. (2014). Tokoferole i tokotrienole jako witamina E. Chemik.

[177] Rizvi S., Mishra M., Agarwal R., Rahman Q. (2014). The role of vitamin E in human health and some diseases. Sultan Qaboos Univ. Med. J..

[178] Maes M., Christophe A., Delanghe J., Altamura C., Neels H., Meltzer H.Y. (2000). Lower serum vitamin E concentrations in major depression: Another marker of lowered antioxidant defenses in that illness. J. Affect. Disord..

[179] Gautam M., Agrawal M., Gautam M., Sharma P., Gautam A.S., Gautam S. (2012). Role of antioxidants in generalised anxiety disorder and depression. Indian J. Psychiatry.

[180] Xu Y., Wang C., Klabnik J.J., O’Donnell J.M. (2014). Novel therapeutic targets in depression and anxiety: Antioxidants as a candidate treatment. Curr. Neuropharmacol..

[181] Hall S., Desbrow B., Anoopkumar-Dukie S., Davey A.K., Arora D., McDermott C., Schubert M.M., Perkins A.V., Kiefel M.J., Grant G.D. (2015). A review of the bioactivity of coffee, caffeine and key coffee constituents on inflammatory responses linked to depression. Food Res. Int..

[182] Tchekalarova J., Pechlivanova D., Atanasova T., Yakimova K., Nikolov R. (2011). P.1.d.005 Depressive-like behaviour of Wistar rats exposed to chronic unpredictable stress: Effect of long-term caffeine administration. Eur. Neuropsychopharmacol..

[183] Trebatická J., Ďuračková Z. (2015). Psychiatric disorders and polyphenols: Can they be helpful in therapy?. Oxid. Med. Cell. Longev..

[184] Fang J.-L., Zhang Q., Zhao Y., Li X., Wang Y. (2020). Ameliorative effect of anthocyanin on depression mice by increasing monoamine neurotransmitter and up-regulating BDNF expression. J. Funct. Foods.

[185] Grzelczyk J., Stojakowska A., Walkowiak A., Łuczaj W., Skrzydlewska E. (2021). Evaluation of the inhibition of monoamine oxidase A by bioactive coffee compounds protecting serotonin degradation. Food Chem..

[186] Van Praag H., Kempermann G., Gage F.H. (2007). Plant-derived flavanol (−) epicatechin enhances angiogenesis and retention of spatial memory in mice. J. Neurosci..

[187] Kopeć A., Kopeć T., Pietrzak K., Zduńczyk Z. (2011). Prozdrowotne właściwości resweratrolu. Żywność Nauka Technol. Jakość.

[188] Wang F., Wang J., An J., Yuan G., Hao X., Zhang Y. (2018). Resveratrol ameliorates depressive disorder through the netrin1-mediated extracellular signal-regulated kinase/cAMP signal transduction pathway. Mol. Med. Rep..

[189] Malaguarnera G., Pennisi M., Bertino G., Motta M., Borzi A.M., Vicari E., Bella R., Drago F., Malaguarnera M. (2018). Resveratrol in patients with minimal hepatic encephalopathy. Nutrients.

[190] Witte A.V., Kerti L., Margulies D.S., Floel A. (2014). Effects of resveratrol on memory performance, hippocampal functional connectivity, and glucose metabolism in healthy older adults. J. Neurosci..

[191] Moore A., Beidler J., Hong M.Y. (2018). Resveratrol and depression in animal models: A systematic review of the biological mechanisms. Molecules.

[192] Lachowicz M., Stańczak A., Kołodziejczyk M. (2020). Kurkumina—Naturalny polifenol o wielu właściwościach—Rozwiązania technologiczne wspomagające farmakoterapię. Farm. Pol..

[193] Fusar-Poli L., Vozza L., Gabbiadini A., Vanella A., Concas I., Tinacci S., Petralia A., Signorelli M.S., Aguglia E. (2020). Curcumin for depression: A meta-analysis. Crit. Rev. Food Sci. Nutr..

[194] Adjibade M., Andreeva V.A., Lemogne C., Touvier M., Shivappa N., Hébert J.R., Wirth M.D., Hercberg S., Galan P., Julia C. (2017). The inflammatory potential of the diet is associated with depressive symptoms in different subgroups of the general population. J. Nutr..

[195] Pang C., Yu H., Xie J., Chen Y. (2025). Pro-inflammatory diet and the risk of depression and anxiety: A prospective study based on the dietary inflammatory index. J. Affect. Disord..

[196] Zhai Y., Hu F., Yuan L., Wang L., Ye X., Cao Y., He J., Sun J., Xu F. (2024). Associations between an energy-adjusted inflammatory diet index and incident depression: A cohort study. Br. J. Nutr..

[197] Shivappa N., Schoenaker D.A.J.M., Hebert J.R., Mishra G.D. (2016). Association between inflammatory potential of diet and risk of depression in middle-aged women: The Australian Longitudinal Study on Women’s Health. Br. J. Nutr..

[198] Manzel A., Muller D.N., Hafler D.A., Erdman S.E., Linker R.A., Kleinewietfeld M. (2014). Role of “Western diet” in inflammatory autoimmune diseases. Curr. Allergy Asthma Rep..

[199] Popkin B.M., Corvalan C., Grummer-Strawn L.M. (2020). Dynamics of the double burden of malnutrition and the changing nutrition reality. Lancet.

[200] Thomas-Valdés S., Tostes M.D.G.V., Anunciação P.C., da Silva B.P., Sant’Ana H.M.P. (2017). Association between vitamin deficiency and metabolic disorders related to obesity. Crit. Rev. Food Sci. Nutr..

[201] Cordain L., Eaton S.B., Sebastian A., Mann N., Lindeberg S., Watkins B.A., O’Keefe J., Brand-Miller J. (2005). Origins and evolution of the Western diet: Health implications for the 21st century. Am. J. Clin. Nutr..

[202] Kramer H. (2017). Kidney Disease and the Westernization and Industrialization of Food. Am. J. Kidney Dis..

[203] Wilczyńska A., Modrzewski A. (2019). Fatty Acids in Human Diet and Their Impact on Cognitive and Emotional Functioning. The Role of Functional Food Security in Global Health.

[204] Zemdegs J., Quesseveur G., Jarriault D., Pénicaud L., Fioramonti X., Guiard B.P. (2016). High-fat diet-induced metabolic disorders impairs 5-HT function and anxiety-like behavior in mice. Br. J. Pharmacol..

[205] Li Y., Cheng Y., Zhou Y., Du H., Zhang C., Zhao Z., Chen Y., Zhou Z., Mei J., Wu W. (2022). High fat diet-induced obesity leads to depressive and anxiety-like behaviors in mice via AMPK/mTOR-mediated autophagy. Exp. Neurol..

[206] Jacka F.N., Pasco J.A., Mykletun A., Williams L.J., Hodge A.M., O'Reilly S.L., Nicholson G.C., Kotowicz M.A., Berk M. (2010). Association of Western and traditional diets with depression and anxiety in women. Am. J. Psychiatry.

[207] Lopresti A.L. (2020). The Effects of Psychological and Environmental Stress on Micronutrient Concentrations in the Body: A Review of the Evidence. Adv. Nutr..

[208] Berding K., Vlčková K., Marx W., Schellekens H., Stanton C., Clarke G., Jacka F., Dinan T.G., Cryan J.F. (2021). Diet and the Microbiota-Gut-Brain Axis: Sowing the Seeds of Good Mental Health. Adv. Nutr..

[209] Ohland C.L., Kish L., Bell H., Thiesen A., Hotte N., Pankiv E., Madsen K.L. (2013). Effects of *Lactobacillus helveticus* on murine behavior are dependent on diet and genotype and correlate with alterations in the gut microbiome. Psychoneuroendocrinology.

[210] Pyndt Jørgensen B., Winther G., Kihl P., Nielsen D.S., Wegener G., Hansen A.K., Sørensen D.B. (2015). Dietary magnesium deficiency affects gut microbiota and anxiety-like behaviour in C57BL/6N mice. Acta Neuropsychiatr..

[211] Magnusson K.R., Hauck L., Jeffrey B.M., Elias V., Humphrey A., Nath R., Perrone A., Bermudez L.E. (2015). Relationships between diet-related changes in the gut microbiome and cognitive flexibility. Neuroscience..

[212] Reichelt A.C., Loughman A., Bernard A., Raipuria M., Abbott K.N., Dachtler J., Van T.T.H., Moore R.J. (2020). An intermittent hypercaloric diet alters gut microbiota, prefrontal cortical gene expression and social behaviours in rats. Nutr. Neurosci..

[213] Burokas A., Arboleya S., Moloney R.D., Peterson V.L., Murphy K., Clarke G., Stanton C., Dinan T.G., Cryan J.F. (2017). Targeting the microbiota-gut-brain axis: Pre-biotics have anxiolytic and antidepressant-like effects and reverse the impact of chronic stress in mice. Biol. Psychiatry..

[214] Dipnall J.F., Pasco J.A., Meyer D., Berk M., Williams L.J., Dodd S., Jacka F.N. (2015). The association between dietary patterns, diabetes and depression. J. Affect. Disord..

[215] Hu D., Cheng L., Jiang W. (2019). Sugar-sweetened beverages consumption and the risk of depression: A meta-analysis of observational studies. J. Affect. Disord..

[216] Puchalska P., Crawford P.A. (2017). Multi-dimensional Roles of Ketone Bodies in Fuel Metabolism, Signaling, and Therapeutics. Cell Metab..

[217] Longo R., Peri C., Cricrì D., Coppi L., Caruso D., Mitro N., De Fabiani E., Crestani M. (2019). Ketogenic Diet: A New Light Shining on Old but Gold Biochemistry. Nutrients.

[218] Capozzi M.E., Coch R.W., Koech J., Astapova I.I., Wait J.B., Encisco S.E., Douros J.D., El K., Finan B., Sloop K.W. (2020). The Limited Role of Glucagon for Ketogenesis During Fasting or in Response to SGLT2 Inhibition. Diabetes.

[219] Gupta L., Khandelwal D., Kalra S., Gupta P., Dutta D., Aggarwal S. (2017). Ketogenic diet in endocrine disorders: Current perspectives. J. Postgrad. Med..

[220] Hui S., Cowan A.J., Zeng X., Yang L., TeSlaa T., Li X., Bartman C., Zhang Z., Jang C., Wang L. (2020). Quantitative Fluxomics of Circulating Metabolites. Cell Metab..

[221] Calderón N., Betancourt L., Hernández L., Rada P. (2017). A ketogenic diet modifies glutamate, gamma-aminobutyric acid and agmatine levels in the hippocampus of rats: A microdialysis study. Neurosci. Lett..

[222] Norwitz N.G., Sethi S., Palmer C.M. (2020). Ketogenic diet as a metabolic treatment for mental illness. Curr. Opin. Endocrinol. Diabetes Obes..

[223] Kraeuter A.K., Phillips R., Sarnyai Z. (2020). Ketogenic therapy in neurodegenerative and psychiatric disorders: From mice to men. Prog. Neuro-Psychopharmacol. Biol. Psychiatry.

[224] Mohorko N., Černelič-Bizjak M., Poklar-Vatovec T., Grom G., Kenig S., Petelin A., Jenko-Pražnikar Z. (2019). Weight loss, improved physical performance, cognitive function, eating behavior, and metabolic profile in a 12-week ketogenic diet in obese adults. Nutr. Res..

[225] St-Onge M.P., Mikic A., Pietrolungo C.E. (2016). Effects of Diet on Sleep Quality. Adv. Nutr..

[226] Cabrera-Mulero A., Tinahones A., Bandera B., Moreno-Indias I., Macías-González M., Tinahones F.J. (2019). Keto microbiota: A powerful contributor to host disease recovery. Rev. Endocr. Metab. Disord..

[227] Olson C.A., Vuong H.E., Yano J.M., Liang Q.Y., Nusbaum D.J., Hsiao E.Y. (2018). The Gut Microbiota Mediates the Anti-Seizure Effects of the Ketogenic Diet. Cell.

[228] Ari C., Kovács Z., Juhasz G., Murdun C., Goldhagen C.R., Koutnik A.P., Poff A.M., Kesl S.L., D'Agostino D.P. (2016). Exogenous Ketone Supplements Reduce Anxiety-Related Behavior in Sprague-Dawley and Wistar Albino Glaxo/Rijswijk Rats. Front. Mol. Neurosci..

[229] Scolnick B. (2017). Ketogenic diet and anorexia nervosa. Med. Hypotheses.

[230] Zhang Y., Liu C., Zhao Y., Zhang X., Li B., Cui R. (2015). The Effects of Calorie Restriction in Depression and Potential Mechanisms. Curr. Neuropharmacol..

[231] Sussman D., Germann J., Henkelman M. (2015). Gestational ketogenic diet programs brain structure and susceptibility to depression & anxiety in the adult mouse offspring. Brain Behav..

[232] Calabrese L., Frase R., Ghaloo M. (2024). Complete remission of depression and anxiety using a ketogenic diet: Case series. Front. Nutr..

[233] Laurent N. (2024). Retrospective case study: Ketogenic metabolic therapy in the effective management of treatment-resistant depressive symptoms in bipolar disorder. Front. Nutr..

[234] Laurent N., Bellamy E.L., Hristova D., Houston A. (2025). Ketogenic metabolic therapy in the remission of chronic major depressive disorder: A retrospective case study. Front. Nutr..

[235] Garner S., Davies E., Barkus E., Kraeuter A.K. (2024). Ketogenic diet has a positive association with mental and emotional well-being in the general population. Nutrition.

[236] Dagnelie P.C., Mariotti F. (2017). Vegetarian diets: Definitions and pitfalls in interpreting literature on health effects of vegetarianism. Vegetarian and Plant-Based Diets in Health and Disease Prevention.

[237] Fraser G.E. (2009). Vegetarian diets: What do we know of their effects on common chronic diseases?. Am. J. Clin. Nutr..

[238] Haghighatdoost F., Hariri M., Feizi A., Aminianfar A., Surkan P.J., Azadbakht L. (2017). Association of vegetarian diet with inflammatory biomarkers: A systematic review and meta-analysis of observational studies. Public Health Nutr..

[239] Neuhouser M.L. (2019). The importance of healthy dietary patterns in chronic disease prevention. Nutr. Res..

[240] Onvani S., Haghighatdoost F., Surkan P.J., Azadbakht L. (2017). Adherence to the Healthy Eating Index and Alternative Healthy Eating Index dietary patterns and mortality from all causes, cardiovascular disease and cancer: A meta-analysis of observational studies. J. Hum. Nutr. Diet..

[241] Menzel J., Jabakhanji A., Biemann R., Mai K., Abraham K., Weikert C. (2020). Systematic review and meta-analysis of the associations of vegan and vegetarian diets with inflammatory biomarkers. Sci. Rep..

[242] Poleszak E. (2007). Modulation of antidepressant-like activity of magnesium by serotonergic system. J. Neural. Transm..

[243] Haider L.M., Schwingshackl L., Hoffmann G., Ekmekcioglu C. (2018). The effect of vegetarian diets on iron status in adults: A systematic review and meta-analysis. Crit. Rev. Food Sci. Nutr..

[244] Allès B., Baudry J., Méjean C., Mariotti F., Berticat C., Deschamps V., Srour B., Julia C., Galan P., Kesse-Guyot E. (2017). Comparison of sociodemographic and nutritional characteristics between self-reported vegetarians, vegans, and meat-eaters from the NutriNet-Santé study. Nutrients.

[245] McEvoy C.T., Temple N., Woodside J.V. (2012). Vegetarian diets, low-meat diets and health: A review. Public Health Nutr..

[246] Allen L.H. (2008). Causes of vitamin B12 and folate deficiency. Food Nutr. Bull..

[247] Rizzo G., Laganà A.S., Rapisarda A.M.C., La Ferrera G., Condorelli R.A., Buscema M., Giacobbe A., Fichera M., Zummo G., La Vignera S. (2016). Vitamin B_12_ among vegetarians: Status, assessment and supplementation. Nutrients.

[248] Tomova A., Bukovsky I., Rembert E., Yonas W., Alwarith J., Barnard N.D., Kahleova H. (2019). The effects of vegetarian and vegan diets on gut microbiota. Front. Nutr..

[249] Timko C.A., Hormes J.M., Chubski J. (2012). Will the real vegetarian please stand up? An investigation of dietary restraint and eating disorder symptoms in vegetarians versus non-vegetarians. Appetite.

[250] Matta J., Czernichow S., Kesse-Guyot E., Hoertel N., Limosin F., Goldberg M., Zins M., Lemogne C. (2018). Depressive symptoms and vegetarian diets: Results from the Constances cohort. Nutrients.

[251] Li X.-D., Cao H.-J., Xie S.-Y., Li K.-C., Tao F.-B., Yang L.-S., Zhang J.-Q., Bao Y.-S. (2019). Adhering to a vegetarian diet may create a greater risk of depressive symptoms in the elderly male Chinese population. J. Affect. Disord..

[252] Hibbeln J.R., Davis J.M., Steigerwalt S., Egert S., Holub B.J., Salem N. (2018). Vegetarian diets and depressive symptoms among men. J. Affect. Disord..

[253] Ocklenburg S., Borawski J. (2021). Vegetarian diet and depression scores: A meta-analysis. J. Affect. Disord..

[254] Michalak J., Zhang X.C., Jacobi F. (2012). Vegetarian diet and mental disorders: Results from a representative community survey. Int. J. Behav. Nutr. Phys. Act..

[255] Jin Y., Kandula N.R., Kanaya A.M., Talegawkar S.A. (2021). Vegetarian diet is inversely associated with prevalence of depression in middle-older aged South Asians in the United States. Ethn. Health.

[256] Beezhold B.L., Johnston C.S., Daigle D.R. (2010). Vegetarian diets are associated with healthy mood states: A cross-sectional study in Seventh-day Adventist adults. Nutr. J..

[257] Białek-Dratwa A., Stoń W., Staśkiewicz-Bartecka W., Grajek M., Krupa-Kotara K., Kowalski O. (2024). The Psychosocial Aspects of Vegetarian Diets: A Cross-Sectional Study of the Motivations, Risks, and Limitations in Daily Life. Nutrients.

[258] Clarys P., Deliens T., Huybrechts I., Deriemaeker P., Vanaelst B., De Keyzer W., Hebbelinck M., Mullie P. (2014). Comparison of nutritional quality of the vegan, vegetarian, semi-vegetarian, pesco-vegetarian and omnivorous diet. Nutrients.

[259] Medawar E., Huhn S., Villringer A., Witte A.V. (2019). The effects of plant-based diets on the body and the brain: A systematic review. Transl. Psychiatry.

[260] Shen Y.-C., Huang Y.-C., Huang T.-C., Pan W.-H., Chiu Y.-F. (2021). Vegetarian diet is associated with lower risk of depression in Taiwan. Nutrients.

[261] Burkert N.T., Pivovarova-Ramich O., Teichmann A., Schwingshackl L., Schlesinger S., Buyken A.E., Alexy U., Dettling M., Hahn A., Stangl G.I. (2014). Nutrition and health–the association between eating behavior and various health parameters: A matched sample study. PLoS ONE.

